# The Genetic Components of a Natural Color Palette: A Comprehensive List of Carotenoid Pathway Mutations in Plants

**DOI:** 10.3389/fpls.2021.806184

**Published:** 2022-01-06

**Authors:** Prateek Gupta, Joseph Hirschberg

**Affiliations:** Department of Genetics, Alexander Silberman Institute of Life Sciences, The Hebrew University of Jerusalem, Jerusalem, Israel

**Keywords:** carotenoid biosynthesis, MEP pathway, mutants, genetic screens, chemical-genetics

## Abstract

Carotenoids comprise the most widely distributed natural pigments. In plants, they play indispensable roles in photosynthesis, furnish colors to flowers and fruit and serve as precursor molecules for the synthesis of apocarotenoids, including aroma and scent, phytohormones and other signaling molecules. Dietary carotenoids are vital to human health as a source of provitamin A and antioxidants. Hence, the enormous interest in carotenoids of crop plants. Over the past three decades, the carotenoid biosynthesis pathway has been mainly deciphered due to the characterization of natural and induced mutations that impair this process. Over the year, numerous mutations have been studied in dozens of plant species. Their phenotypes have significantly expanded our understanding of the biochemical and molecular processes underlying carotenoid accumulation in crops. Several of them were employed in the breeding of crops with higher nutritional value. This compendium of all known random and targeted mutants available in the carotenoid metabolic pathway in plants provides a valuable resource for future research on carotenoid biosynthesis in plant species.

## Introduction

Isoprenoids represent the most functional and diverse class of naturally occurring metabolites present in all organisms. Carotenoids, the largest group of natural pigments, belong to a subgroup of isoprenoid-derived compounds (Maoka, 2020). In plants, carotenoids play diverse functions, first and foremost in photosynthesis as accessory light-harvesting pigments and photoprotectants, and as precursors for the hormones abscisic acid (ABA), strigolactones (Ruiz-Sola and Rodríguez-Concepción, 2012), and other growth regulators (Wang et al., 2020c). In addition, carotenoids play secondary roles in providing distinctive hues and colors to flowers and fruits. Carotenoids’ significance is not limited to plants; their contributions to human health as antioxidants and provitamin A make them indispensable in our diet (Rao and Rao, 2007; Meléndez-Martínez et al., 2021).

Isoprenoid biosynthesis in plants occurs in the cytosol and plastids by the mevalonic acid (MVA) and methylerythritol 4-phosphate (MEP) pathways, respectively. The MVA pathway provides the isopentenyl diphosphate (IPP) precursor for synthesizing sterols, terpenoids, and brassinosteroids. In contrast, the plastidial MEP pathway supplies IPP and dimethylallyl diphosphate (DMAPP) necessary for synthesizing tocopherols, chlorophylls, carotenoids, gibberellic acids, many other terpenoids.

**Figure 1:**
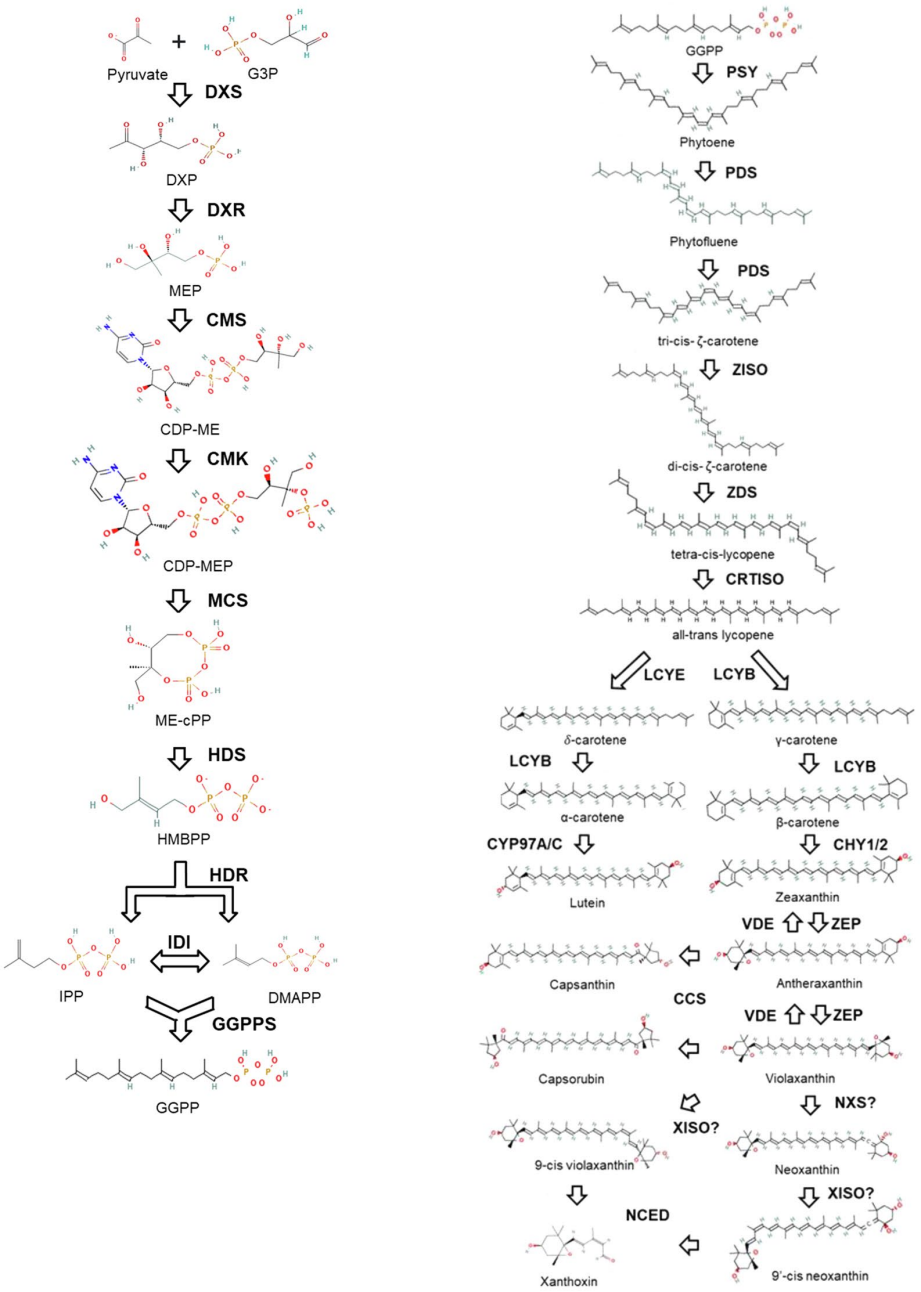
Schematic representation of MEP and carotenoid biosynthesis pathway. Name of the enzymes are highlighted in bold. G3P, Glyceraldehyde 3-phosphate; DXP, 1-deoxy-D-xylulose-5-phosphate; MEP, 2-C-Methyl-D-erythritol 4-phosphate; CDP-ME, 4-diphosphocytidyl-2-C-methyl- D-erythritol; CDP-MEP, 4-diphosphocytidyl-2-C-Methyl-D-erythritol 2-phosphate; ME-cPP, 2-C-methyl-D-erythritol 2,4-cyclodiphosphate; HMBPP, 1-hydroxy-2-methyl-2-(E)-butenyl 4-diphosphate; IPP, isopentenyl diphosphate; DMAPP, dimethylallyl diphosphate; GGPP, geranylgeranyl diphosphate; DXS, 1-deoxy-D-xylulose-5-phosphate synthase; DXR, DXP reductoisomerase; CMS, 4-diphosphocytidyl-2-C-methyl-D-erythritol synthase; CMK, 4-diphosphocytidyl-2-C-methyl-D-erythritol kinase; MCS, 2-C-methyl-D-erythritol 2,4-cyclodiphosphate (ME-cPP) synthase; HDS, 1-hydroxy-2-methyl-2-(E)-butenyl-4-diphosphate synthase; HDR, 1-hydroxy-2-methyl-butenyl 4-diphosphate reductase; IDI, isopentenyl diphosphate isomerase; GGPPS, GGPP synthase; PSY, phytoene synthase; PDS, phytoene desaturase; ZISO, ζ-carotene isomerase; ZDS, ζ-carotene desaturase; CRTISO, carotenoid isomerase; LCYB, lycopene β-cyclase; LCYE, lycopene epsilon-cyclase; CYP97A/C, cytochrome P450 enzymes; CHY1/2, carotene hydroxylase; VDE, violaxanthin deepoxidase; ZEP, zeaxanthin epoxidase; NXS, neoxanthin synthase; CCS, capsanthin-capsorubin synthase; NCED, 9-cis-epoxycarotenoid dioxygenase; XISO, xanthophyll isomerase.

Carotenoid biosynthesis and metabolism have been extensively studied due to their essential roles in plant development and physiology. In the last three decades, all the essential enzymes and genes of the MEP and carotenoid biosynthetic pathways have been identified (Cunningham and Gantt, 1998; Hirschberg, 2001; Phillips et al., 2008b; Cordoba et al., 2009; Ruiz-Sola and Rodríguez-Concepción, 2012; Nisar et al., 2015; Figure 1). Most of the genes in these pathways have been characterized using mutants. Collections of chemically mutagenized plants and transgenic insertion mutations have been the mainstay to obtain mutations in the carotenoid biosynthesis in plants. Molecular characterization of the genes employed transgenic approaches like gene silencing and over-expression. More recently, gene-specific mutations have been created using CRISPR (Clustered Regularly Interspaced Short Palindromic Repeats) along with Cas editing systems.

Mutations that alter metabolism have been widely used in biological research. The isolation and characterization of mutants with altered biochemical properties enabled the discovery of new enzymes and helped decipher new biosynthetic pathways (Beadle, 1945). Many mutations that alter carotenoids in plants have been described over the years. This review presents an overview of the natural and induced mutants available in the carotenoid biosynthesis metabolic pathways in plants.

## Mep Pathway – the Backbone

In plants, the building blocks for isoprenoid biosynthesis and provide the substrate for carotenoid biosynthesis, IPP and DMAPP, are synthesized by MEP (“non-mevalonate”) pathway (Chappell, 1995). The MEP pathway starts with the condensation of pyruvate and glyceraldehyde 3-phosphate (Rodríguez-Concepción and Boronat, 2002). It comprises seven enzymatic steps starting from pyruvate and D-glyceraldehyde-3-phosphate to produce IPP and DMAPP. The first rate-limiting step in the “non-mevalonate” MEP pathway is catalyzed by the 1-deoxy-D-xylulose-5-phosphate synthase (DXS) to form 1-deoxy-D-xylulose-5-phosphate (DXP) from the condensation of D-glyceraldehyde-3-phosphate and pyruvate (Lichtenthaler, 1999). The first mutant reported was the *cla1-1* (*cloroplastos alterados*) isolated from the T-DNA-generated library of *Arabidopsis thaliana in* the *CLA1* gene (Mandel et al., 1996). Later on, it was established that the *CLA1* gene encodes the first step in the MEP pathway, and it is the same as *DXS* (Estevez et al., 2000). After that there are three more mutants that are allelic to *cla1-1* are reported in Arabidopsis *viz.*, *chs5* (Araki et al., 2000)*, ivr111* (Crowell et al., 2003), and *cla1-2* (Gutiérrez-Nava et al., 2004)*. cla1* mutant exhibits lethal albino phenotype, whereas *ivr111* and *chs5* show variegated phenotype and temperature-sensitive chlorotic phenotype, respectively. Like the *cla1* mutant, white-lethal-seeding-2297 (wls-2297) was isolated from the T-DNA mutant collection of tomatoes, which also exhibit lethal albino phenotype (García-Alcázar et al., 2017).

The second reaction, which involves the reduction and rearrangement of DXP into 2-C-Methyl-D-erythritol 4-phosphate (MEP), is catalyzed by DXP reductoisomerase (DXR) enzyme. Four T-DNA insertion mutant lines are available for the *DXR* gene, two each in Arabidopsis and rice, all displaying the albino phenotype (Budziszewski et al., 2001; Jung et al., 2008; Xing et al., 2010). In the third step of the MEP pathway, 4-diphosphocytidyl-2-C-methyl-D-erythritol synthase (CMS or IspD), converts MEP into 4-diphosphocytidyl-2-C-Methyl-D-erythritol (CDP-ME) by adding CTP to it. Two T-DNA insertion mutants, *ispD-1* and *ispD-2*, were isolated from Arabidopsis with the albino phenotype (Hsieh et al., 2008). However, two weak alleles, *isp1-1* and *isp 1–2*, have also been isolated from EMS population of Arabidopsis, exhibiting high chlorophyll fluorescence phenotype (Hojo et al., 2005).

The fourth enzyme in the MEP pathway leads to the formation of 4-diphosphocytidyl-2-C-methyl- D-erythritol 2-phosphate (CDP-MEP) from CDP-ME in an ATP dependent manner. This reaction is catalyzed by 4-diphosphocytidyl-2-C-methyl-D-erythritol kinase (CMK or IspE), encoded by the gene IspE. *IspE-1,* a T-DNA insertion mutant in Arabidopsis, and *green-revertible yellow leaf* (*gry340*), an EMS mutant in rice, display albino and virescent phenotype, respectively, at the early stages of plant development (Hsieh et al., 2008; Chen et al., 2018b). The enzyme 2-C-methyl-D-erythritol 2,4-cyclodiphosphate (ME-cPP) synthase (MCS or IspF), catalyzes the cyclization of CDP-MEP into 2-C-methyl-d-erythritol 2,4-cyclodiphosphate (ME-cPP), is the fifth step in the MEP pathway. In Arabidopsis, two T-DNA null mutant lines are available, *ispF-1* and GT-0946, displaying the lethal albino phenotype. By contrast, in rice, an EMS mutant in this gene, 505ys is reported exhibiting yellow-green leaf phenotype throughout plant development (Budziszewski et al., 2001; Hsieh and Goodman, 2006; Huang et al., 2018a).

The penultimate step of the MEP pathway is carried out by 1-hydroxy-2-methyl-2-(E)-butenyl-4-diphosphate synthase (HDS or IspG), which converts ME-cPP to 1-hydroxy-2-methyl-2-(E)-butenyl 4-diphosphate (HMBPP). In Arabidopsis, there are three allelic mutations in HDS- *clb1-1* (*chloroplast biogenesis*), a T-DNA insertion line, and *clb1-2* and *csb3* (*constitutive subtilisin3*) isolated from EMS population (Gutiérrez-Nava et al., 2004; Gil et al., 2005). The *clb* null mutants show albino phenotypes, whereas *csb* is a partial loss-of-function mutation. *Seed carotenoid deficient* (*scd-1* and *scd-2*), *lemon white* (*lw*), *viviparous12* (*vp12*) belong to the category of spontaneous/natural mutations reported in the *HDS* gene in maize all show a characteristic of albino plants with pale-yellow seeds (Zhang et al., 2019a).

The last step in the MEP pathway is 1-hydroxy-2-methyl-butenyl 4-diphosphate reductase (HDR or IspH), which converts HMBPP into both isopentenyl diphosphate (IPP) and dimethylallyl diphosphate (DMAPP) at a ratio of 6:1 (Rohdich et al., 2006; Tritsch et al., 2010). The null T-DNA insertion mutant in Arabidopsis, *IspH-1,* and the EMS-induced mutant, *clb6-1*, showed albino phenotypes (Guevara-García et al., 2005; Hsieh and Goodman, 2005), whereas an EMS mutant in maize, *zebra7*, displayed transverse yellow/green striped leaves at the early stages of development (Lu et al., 2012). The first-ever report of CRISPR/Cas editing in the MEP pathway was reported in the *IspH* gene of *N. benthamiana,* where newly developed leaves showed photobleached phenotype (Yin et al., 2015). Although the MEP pathway leads to the synthesis of both IPP and DMAPP, the 6:1 ratio of these compounds may limit carotenoid biosynthesis (Pankratov et al., 2016). The interconversion of IPP and DMAPP is mediated by isopentenyl diphosphate isomerase (IDI). Plants have two IDI enzymes, IDI1 and IDI2, targeted to different cell compartments. T-DNA single null mutants in Arabidopsis (*idi1-1, idi1-2, idi2-1, Atipi1,* and *Atipi2*) either in IDI1 or IDI2 do not exhibit an apparent phenotype while the double mutants are either not viable or show severe pleiotropic phenotype (Okada et al., 2008; Phillips et al., 2008a). In tomato, four allelic mutants (three EMS-generated and one spontaneous) of the plastidial enzyme IDI1 exists- *fcd1-1*, *fcd1-2*, *fcd1-3* and *fcd^at^* (fruit carotenoid deficient1). Contrary to the Arabidopsis *IDI1* mutants, *fcd1* mutants showed a reduced concentration of carotenoids in cotyledon, fruits, and flowers (Pankratov et al., 2016).

Synthesis of Geranylgeranyl diphosphate (GGPP) from IPP and DMAPP, is a three-step head-to-tail condensation process catalyzed by GGPP synthase (GGPPS). GGPP serves as the central precursor for carotenoids and terpenoids, tocopherols, chlorophyll side chains, gibberellic, and plastoquinones. There are 11 isoforms of GGPPS in Arabidopsis, from which T-DNA mutants of *ggpps2*, *ggpps6*, *ggpps7*, *ggpps8,* and *ggpps10* did not show any developmental defect (Ruiz-Sola et al., 2016), T-DNA mutants in *GGPPS11* (*ggpps11-2*, *ggpps11-3*, *ggpps11-4*) showed albino-lethal phenotype highlighting its importance in plant development. There are two more weak allelic mutations, namely, *ggpps11-1* and *ggpps11-5*, which showed variegated and paler leaf phenotype (Ruppel et al., 2013). Like Arabidopsis, multiple isoforms of GGPPS exist in tomato and CRISPR mutants for plastidial *GGPPS2* and *GGPPS3* were generated (Barja et al., 2021). Mutants impaired in *GGPPS3* (*slg3-1* and *slg3-2*) but not in *GGPPS2* (*slg2-1* and *slg2-2*) showed lower levels of photosynthetic pigments, whereas double mutants were not viable. The list of mutants described in the MEP pathway is presented in Table 1.

**Table 1:** List of mutants identified in MEP pathway.

Gene	Insertional mutagenesis	CRISPR/Cas	Induced mutagenesis	Spontaneous/natural mutation
*DXS*	*cla1-1* (At4g15560; Arabidopsis; Mandel et al., 1996; Estevez et al., 2000) *wls-2297* (Solyc01g067890) (Tomato; García-Alcázar et al., 2017)		*chs5, lvr111* (EMS), *cla1-2* (At4g15560; Ethylenimine; Arabidopsis) (Araki et al., 2000; Crowell et al., 2003; Gutiérrez-Nava et al., 2004)	
*DXR*	GK_215C01, 4036 (At5g62790; Arabidopsis; Budziszewski et al., 2001; Xing et al., 2010) 1A-14224 and 1C-03301 (Os01g01710) (Rice) (Jung et al., 2008)			
*CMS/IspD*	*ispD-1*, *ispD-2* (At2g02500; Arabidopsis) (Hsieh et al., 2008)		*isp1-1*, *isp1-2* (At2g02500; EMS; Arabidopsis) (Hojo et al., 2005)	
*CMK/IspE*	*ispE-1* (At2g26930; Arabidopsis; Hsieh et al., 2008)		*gry340* (Os01g58790; EMS; Rice; Chen et al., 2018b)	
*MCS/IspF*	*IspF-1,* GT0946 (At1g63970; Arabidopsis; Budziszewski et al., 2001; Hsieh and Goodman, 2006)		*505ys* (Os02g45660; EMS; Rice; Huang et al., 2018a)	
*HDS/IspG*	*clb4-2* (At5g60600; Arabidopsis; Gutiérrez-Nava et al., 2004)	scd (Maize; GRMZM2G137409; Zhang et al., 2019a)	*Clb4-1, csb3* (At5g60600; EMS; Arabidopsis; Gutiérrez-Nava et al., 2004; Gil et al., 2005)	*scd, lw2-vp12, scd-1, scd-2* (GRMZM2G137409; Maize; Zhang et al., 2019a)
*HDR/IspH*	*ispH-1* (At4g34350; Arabidopsis; Hsieh and Goodman, 2005)	Tobacco (Yin et al., 2015)	*clb6-1* (At4g34350; EMS; Arabidopsis; Guevara-García et al., 2005) *zebra7* (GRMZM2G027059; EMS; Maize; Lu et al., 2012)	
*IDI*	*idi1-1, idi1-2, Atipi1* (At5g16440) *idi2-1, Atipi2* (At3g02780; Arabidopsis) (Okada et al., 2008; Phillips et al., 2008a)		*fcd 1–1, fcd 1–2, fcd 1–3* (Solyc04g056390; EMS; Tomato; Pankratov et al., 2016)	*fcd^at^* (Solyc04g056390; Tomato; Pankratov et al., 2016)
*GGPPS*	*ggpps2* (At2g18620)*, ggpps6* (At3g14530)*, ggpps7* (At3g14550)*, ggpps8* (At3g20160)*, ggpps10* (At3g32040)*, ggpps11-3, ggpps11-4, ggpps11-5* (At4g36810; Arabidopsis) (Ruppel et al., 2013; Ruiz-Sola et al., 2016)	*slg2-1, slg2-2* (Solyc04g079960) *slg3-1, slg3-2* (Solyc02g085700) (Tomato; Barja et al., 2021)	*ggps11-1* (At4g36810; EMS; Arabidopsis; Ruppel et al., 2013)	

## Carotenoid Biosynthesis – the Central Pathway

### Phytoene Synthesis – The Bottleneck

The first committed step in C40 carotenoid biosynthesis is the head-to-head condensation of two GGPP molecules by the key regulatory enzyme phytoene synthase (PSY) to form 15-*cis* phytoene. While many plant species have a single *PSY* gene, some species contain three isoforms. In tomato, for example, *PSY1* functions in chromoplasts, *PSY2* in chloroplasts, and *PSY3* in root plastids. Defects in the functional copy of *PSY1* lead to yellow fruit lacking carotenoids. Several loss-of-function mutations in the PSY1 gene of tomato (genetic locus *r*) have been isolated (*r^2997^*, *r^3756^*, *r^y^*, *yft2*, PI114490; Fray and Grierson, 1993; Yuan et al., 2008; Gady et al., 2012; Kachanovsky et al., 2012; Chen et al., 2019). Likewise, CRISPR mutants have also been generated for *PSY1* in tomato and maize, *PSY* in wheat, and both *PSY1* and *PSY2* in carrots (Zhu et al., 2016; Dahan-Meir et al., 2018; D’Ambrosio et al., 2018; Oleszkiewicz et al., 2021; Zhang et al., 2021). Reduced accumulation of carotenoids characterize CRISPR mutants in wheat, while the albino plants and white seeds are observed in maize (Zhu et al., 2016; Zhang et al., 2021). In carrots, *PSY1* and *PSY2* mutants display pale orange to yellow pigmentation in callus, with *PSY2* is critical for carotenogenesis in roots (Oleszkiewicz et al., 2021). Spontaneous loss-of-function mutant lines of *PSY* exist in pepper, maize, loquat, and poppy, leading to reduced levels of carotenoids, while gain-of-function mutant exists in cassava leading to enhanced carotenoid production (Robertson and Anderson, 1961; Buckner et al., 1996; Kim et al., 2010; Welsch et al., 2010; Fu et al., 2014; Jeong et al., 2019; Pollack et al., 2019). Eight missense mutations in *PSY* were reported in melon. However, no phenotype has been recorded in these lines (Vicente-Dólera et al., 2014).

### Desaturation and Isomerization – The Poly-*Cis* Pathway

Conversion of 15-*cis*-phytoene to all-*trans* lycopene entails four double bond desaturations (dehydrogenations) and three *cis-trans* isomerizations. Intermediate carotenes in this pathway are all *cis*-configured (Isaacson et al., 2004). Phytoene desaturation is catalyzed by phytoene desaturase (PDS) in a two-step process leading to the production of phytofluene followed by 9,15,9′-tri-*cis*-ζ-carotene. Loss-of-function mutations in *PDS* resulted in albinism and dwarf phenotypes in the Arabidopsis T-DNA insertion mutant *pds3*, reduced carotenoid content in petals of *B. napus* in the *ywf* (*yellow-white flower*) mutant, white seed, and premature seed germination in the maize *vp5* (*viviparous5*) mutant and the lethal albino phenotype in *phs1* (*pre-harvest sprouting1*) in rice (Hable et al., 1998; Qin et al., 2007; Fang et al., 2008; Zhao et al., 2021). *PDS* has been used as a reporter gene in transient gene silencing studies due to the bleached leaves phenotype visible to the naked eye (Kumagai et al., 1995; Ratcliff et al., 2001). It should be noted that PDS, the first gene identified in the carotenoid biosynthesis pathway in plants, was initially detected due to mutations that conferred resistance to the “bleaching herbicide” norflurazon (Chamovitz et al., 1990). Several missense mutations in the cyanobacterial PDS that alter conserved amino acid residues of the enzyme in plants lower the binding affinity of norflurazon and thus confer herbicide resistance (Chamovitz et al., 1993; Wagner et al., 2002). Additional mutations in PDS from algae and plants that confer resistance to “bleaching herbicides” have been reported (Michel et al., 2004; Suarez et al., 2014; Dang et al., 2018; Taparia et al., 2019).

The first isomerization step by ζ-carotene isomerase (ZISO) converts 9,15,9′-tri-*cis*-ζ-carotene to 9,9′-di-*cis*-ζ-carotene. In photosynthetically active chloroplasts, this isomerization can be mediated by light. However, ZISO is critical in chromoplasts and root plastids. The tillering mutants *t20* and *htd12* in rice, which are impaired in ZISO, display a delayed greening phenotype similar to the T-DNA insertion and EMS mutants in ZISO of Arabidopsis (*zic*; Chen et al., 2010; Liu et al., 2020a; Zhou et al., 2021). Other mutants of *ZISO* in tomato (*Zeta*, *e2803*), Arabidopsis (*ziso-155*), maize (*y9*), and orange (*pinalate*), accumulates high levels of phytoene, phytofluene, and ζ –carotene in fruits and seeds (Li et al., 2007; Kachanovsky et al., 2012; Rodrigo et al., 2019; Cazzonelli et al., 2020; Gupta et al., 2021).

Zeta-carotene desaturase (ZDS) catalyzes the final desaturation steps, converting 9,9′-di-*cis*-ζ-carotene to 9,9′,11,11′-tetra-*cis* lycopene (“prolycopene”). Several *ZDS* mutants have been reported and characterized in plants. The strong-mutant alleles *spontaneous cell death* (*spc1-2*) and chloroplast biogenesis (*clb5-1, clb5-2*) in Arabidopsis display albino-lethal phenotype while weak alleles *spc1-3* and *clb5-3* have mild phenotype (Dong et al., 2007; Avendaño-Vázquez et al., 2014). Null mutants in maize (*alb1, vp-wl2, vp9*), rice (*ale1, phs2*) and sunflower (*nd-1*) also display albino-lethal phenotypes (Conti et al., 2004; Fang et al., 2008, 2019; Chen et al., 2017; Wang et al., 2020b).

In the second isomerization reaction, tetra-*cis* lycopene is converted to all-*trans* lycopene by the enzyme carotenoid isomerase (CRTISO). *CRTISO* was first characterized in tomato *tangerine* (*t^3183^, t^mic^*) and Arabidopsis *ccr2* mutants, which accumulate tetra-*cis* lycopene in fruits and seedlings, imparting the orange-yellow color to the fruits and seedlings, respectively (Isaacson et al., 2002; Park et al., 2002). Later on, *CRTISO* mutants were identified in rice (*phs3, zebra2, mit3*), melon (*yofi*), orange flower calendula, and orange color Chinese cabbage, marked with an accumulation of tetra-*cis* lycopene and reduced lutein content (Fang et al., 2008; Kishimoto and Ohmiya, 2012; Galpaz et al., 2013; Zhao et al., 2014; Su et al., 2015; Liu et al., 2018). CRISPR mutants of *CRTISO* were also reported in Chinese kale and tomato with similar phenotypes (Dahan-Meir et al., 2018; Sun et al., 2020a; Lakshmi Jayaraj et al., 2021).

### Lycopene Cyclization – The Branching Point

Cyclization of lycopene bifurcates the pathway into two branches, the β-β branch leading to violaxanthin and neoxanthin and the ε-β branch leading to lutein. In the β-β branch, two β-rings are formed at both ends of the lycopene molecule by the enzyme lycopene β-cyclase (LCYB, CRT-L) to produce β-carotene. In contrast, in the ε-β branch, lycopene is first cyclized by lycopene epsilon-cyclase (LCYE) and then by LCYB to produce α-carotene. Some plants have several lycopene beta-cyclase paralogs, which are differentially expressed. The lycopene beta-cyclases expressed in chloroplast-containing tissues (designated as *LCYB*) are indispensable for plant growth, whereas beta-cyclases that predominantly function in chromoplasts-containing tissues like fruits and flowers (designated as *CYCB* in tomato) solely affect the colors. In tomato, two types of mutations exist in the *CYCB*, *Beta* (*B*). A dominant mutation that leads to high β-carotene in fruit, and *old-gold* (*B^og^*) and *beta-crimson* (*B^c^*), which are recessive loss-of-function mutants with increased lycopene and reduced β-carotene in fruits (Ronen et al., 2000). Similar mutations in *lcyb* of papaya, maize (*lcyb-m2.1*), and rice (*phs4*) accumulate lycopene leading to red-fleshed papaya fruit, slightly pink kernels in maize, and pink-embryo seeds in rice (Fang et al., 2008; Bai et al., 2009; Devitt et al., 2010). The Arabidopsis *suppressor of zeaxanthin-less1* (*szl1*) mutant, which has a point mutation in *LCYB*, accumulates more lutein and small amounts of xanthophyll-cycle pigments (Li et al., 2009). A loss-of-function mutation in *LCYE* in the Arabidopsis mutant *lut2* eliminated lutein and increased the concentration of β-carotene along with xanthophyll-cycle pigments (Pogson et al., 1996). A dominant mutation, *DEL*, in the gene LCY-E of tomato increases the expression of lycopene ε-cyclase in the fruits, which accumulate δ-carotene (Ronen et al., 1999). Multiplex CRISPR/Cas9-based genome editing has also been done in *LCYB1*, *LCYB2*, *CYCB*, *LCYE,* and *SGR1* genes to achieve lycopene enriched tomatoes (Li et al., 2018b). CRISPR and EMS mutants of *LCYE* in banana and wheat accumulated β-carotene in fruits and leaves, respectively (Richaud et al., 2018; Kaur et al., 2020).

### Hydroxylation – The Primary Route for Xanthophyll Biosynthesis

The biosynthesis of xanthophylls from α-carotene and β-carotene requires ring-specific hydroxylations. The ε- and β-ring hydroxylation of α-carotene are catalyzed by heme-containing cytochrome P450 enzymes (CYP97A and CYP97C), yielding lutein. The non-heme β-carotene hydroxylases BCH1 and BCH2, catalyze the β-ring hydroxylation of β-carotene to produce zeaxanthin. *BCH1/2* mutations have been reported in tomato, Arabidopsis, rice, pepper, and maize. The mutations *white flower* (*wf*) in tomato and *E172-3* in pepper in the genes *CrtR-b2/BCH2* abolish xanthophyll accumulation in flowers of tomato and pepper fruits (Galpaz et al., 2006; Borovsky et al., 2013). Similarly, *dsm2* mutants in rice and *crtRB1* mutants in maize reduce the accumulation of zeaxanthin and increase β-carotene content (Du et al., 2010; Yan et al., 2010). However, single T-DNA mutants of *BCH1* (*b1*) and *BCH2* (*b2*) in Arabidopsis do not have a significant impact on carotenoid composition in leaves, and the double mutant *b1b2* only show a partial reduction in β-carotene derived xanthophylls (Tian et al., 2003).

Mutations in *CYP97A* in Arabidopsis (*lut5*), rice (*cyp94a-4*), and orange carrots increase the level of α-carotene and reduce lutein concentrations (Kim and DellaPenna, 2006; Lv et al., 2012; Arango et al., 2014). The Arabidopsis *lut1* mutation in *CYP97C* is marked by the absence of lutein, the accumulation of zeinoxanthin, and xanthophyll-cycle pigments (Pogson et al., 1996; Tian et al., 2004). Double, triple, and quadruple carotene hydroxylase mutations exist in Arabidopsis. One such mutant, *nox* (*no xanthophyll*), obtained by combining all the four hydroxylase mutants, is devoid of xanthophylls and predominantly accumulates α- and β-carotene (Dall’Osto et al., 2013).

The last steps in the β-branch of xanthophyll biosynthesis convert zeaxanthin to violaxanthin by the zeaxanthin epoxidase (ZEP) followed by neoxanthin synthesis through an unknown reaction. These xanthophylls, together with lutein, are components of (LHCs). Zeaxanthin plays an essential role in excess energy dissipation that protects the light-harvesting complexes (LHCs). However, since it is not a constituent of the LHC, it is rapidly synthesized under light conditions by deeopxidation of violaxanthin catalyzed by the violaxanthin deepoxidase (VDE) with antheraxanthin (A) as an intermediate. The interconversion of zeaxanthin to violaxanthin and vice versa is known as the xanthophyll (or violaxanthin) cycle. Mutations in *ZEP* in *N. plumbaginifolia* (*aba2*), Arabidopsis (*aba1 and npq2*), and rice (*Osaba1*) show accumulation of zeaxanthin and absence of violaxanthin and neoxanthin in mutant leaves (Duckham et al., 1991; Rock and Zeevaart, 1991; Marin et al., 1996; Niyogi et al., 1998; Agrawal et al., 2001; Gonzalez-Jorge et al., 2016). However, *ZEP* mutants in tomato (*hp3*), pepper, and *B. napus* display differential accumulation of carotenoids in fruits, flowers, and leaves (Galpaz et al., 2008; Liu et al., 2020b; Lee et al., 2021). The *hp3* mutation increases total carotenoids in fruits accompanied by an atypical accumulation of zeaxanthin and eliminates violaxanthin and neoxanthin in leaves (Galpaz et al., 2008). In pepper and *B. napus*, mutations in ZEP increase the level of zeaxanthin at the expense of violaxanthin in fruits and flowers, with no change in leaf carotenoids. The only mutation known in VDE is the Arabidopsis *npq1*, where the mutant cannot convert violaxanthin to antheraxanthin and zeaxanthin in excessive light, thus resulting in high-light sensitive plants (Niyogi et al., 1998). The last step in the carotenoid biosynthetic pathway is the synthesis of neoxanthin from violaxanthin, which takes place in a poorly understood enzymatic reaction. Two mutants that lack neoxanthin in tomato (*neoxanthin-deficient1, nxd1*) and Arabidopsis (*ABA-deficient4, aba4*) were identified. However, the exact role of NXD1 and ABA4 proteins remained unknown as their exact enzymatic activities have not been established (North et al., 2007; Neuman et al., 2014; Perreau et al., 2020). Since NXD1 exists in the cytoplasm while ABA4 is found within plastids, it is likely that the latter is involved in this reaction (Perreau et al., 2020). An additional enzyme in peppers (Capsicum species), capsanthin-capsorubin synthase (CCS), converts antheraxanthin and violaxanthin to capsanthin and capsorubin, respectively (Bouvier et al., 1994). There are many allelic variations present in the *ccs* gene in pepper which includes structural variation in both promoter and coding region, early translational termination, frame-shift mutations, and missense mutations imparting different hues to fruit color in non-red pepper accessions (Lefebvre et al., 1998; Popovsky and Paran, 2000; Ha et al., 2007; Guzman et al., 2010; Li et al., 2013b; Jeong et al., 2019). The mutants available in the carotenoid biosynthetic pathway are listed in Table 2.

**Table 2:** List of mutants identified in carotenoid biosynthetic pathway.

Gene	Insertional mutagenesis	CRISPR	Induced mutagen	Spontaneous/natural mutation
*PSY*		*Psy1* (Solyc03g031860; Tomato; Dahan-Meir et al., 2018; D’Ambrosio et al., 2018) Maize (GRMZM2G300348; Zhu et al., 2016) Wheat (Zhang et al., 2021) Carrot *psy1* (GeneBank Gene ID: 108227339), *psy2* (GeneBank Gene ID: 108214656) (Oleszkiewicz et al., 2021)	line 5,381 and 1804, *r3756* (Solyc03g031860; EMS; Tomato; Gady et al., 2012; Kachanovsky et al., 2012) melon (EMS; *Cucurbita pepo*; Vicente-Dólera et al., 2014)	PI114490, *r, r^y^, yft2* (Solyc03g031860; Tomato; Fray and Grierson, 1993; Yuan et al., 2008; Chen et al., 2019) Pepper *PSY1* (CAA48155, CA04g04080), *PSY2* (CA02g20350) (Kim et al., 2010; Jeong et al., 2019) *pas-8,549, w^mut^, y1-2053* (Maize; Robertson and Anderson, 1961; Buckner et al., 1996) white-fleshed (Loquat) (Fu et al., 2014) white petal california poppy (Pollack et al., 2019) Cassava PSY2 (GU111720) (Welsch et al., 2010)
*PDS*	*pds3* (At4g14210; Arabidopsis; Qin et al., 2007) *phs1* (Os03g08570; Rice; Fang et al., 2008)	Tomato (Solyc03g123760) (Pan et al., 2016; Li et al., 2018a) Rice *OsPDS* (Os03g08570) *OsPDS1* (Os03g0184000) (Shan et al., 2013; Zhang et al., 2014; Ishizaki, 2016; Banakar et al., 2019) Tobacco (Nekrasov et al., 2013; Li et al., 2013a; Ali et al., 2015, 2018; Gao et al., 2015; Yin et al., 2015; Endo et al., 2016; Chen et al., 2018a; Ren et al., 2019a, 2021; Schmitz et al., 2020) Arabidopsis *pds3* (At4g14210) (Li et al., 2013a; Tsutsui and Higashiyama, 2017; Ali et al., 2018; Wolabu et al., 2020) Brassica *BoPDS* (Bol016089) *BoPDS1* (Bol009962) *BaPDS1* (GenBank Accession No. KX426039), *BaPDS2* (GenBank Accession No. KX426040) (Murovec et al., 2018; Sun et al., 2018; Ma et al., 2019a,b; Lee et al., 2020) Banana *PDS* (GenBank Accession JQ762260) *MaPDS* (Ma08_g16510) (Hu et al., 2017; Kaur et al., 2018; Naim et al., 2018; Ntui et al., 2020; Wu et al., 2020) Citrus (Jia and Nian, 2014; Jia and Wang, 2014; Jia et al., 2017, 2019; Zhang et al., 2017; Zhu et al., 2019; Dutt et al., 2020) Grape (Nakajima et al., 2017; Ren et al., 2019a,b, 2021) Hop *HlPDS* (NCBI accession number: MT083893) (Awasthi et al., 2021)	yellowish-white flower (EMS; *Brassica napus*) (Zhao et al., 2021)	*vp5* (maize) (Hable et al., 1998)
		Apple *MdPDS* (MD04G0021400) (Nishitani et al., 2016; Osakabe et al., 2018; Charrier et al., 2019) Cassava (Manes.05G193700) (Odipio et al., 2017) Melon *CmPDS* (MELO3C017772.2) (Tian et al., 2017; Hooghvorst et al., 2019) Strawberry *FvPDS* (LG4-gene12690) (Wilson et al., 2019) Hieracium (Henderson et al., 2020) Populus (Fan et al., 2015; Liu et al., 2015; Wang et al., 2020a) Chicory *CiPDS* (GenBank accession MK455771) (Bernard et al., 2019) Yam (Syombua et al., 2021) Carrot (Genbank accession no. XM_017396654.1) (Xu et al., 2019) Rehmannia (Li et al., 2021) Witloof (De Bruyn et al., 2020) Sorghum (Liu et al., 2019) Soybean *GmPDS11* (Glyma.11G253000), *GmPDS18* (Glyma.18G003900) (Du et al., 2016) Tragopogon (Shan et al., 2018) Medicago (Meng et al., 2017; Wolabu et al., 2020) Potato (Wang et al., 2015; Bánfalvi et al., 2020) Hevea (Dai et al., 2021) Wheat (Upadhyay et al., 2013; Howells et al., 2018) Lilium (Yan et al., 2019) Kiwifruit		
		*AcPDS* (Ach19g199631) (Wang et al., 2018) Petunia *PhPDS* (GenBank ID: KP677483) (Zhang et al., 2016)		
*ZISO*	*zic 1–3, zic 1–4, zic 1–5, zic 1–6* (At1g10830) (Arabidopsis) (Chen et al., 2010)	*t20-2, t20-3* (Os12g21710; Rice) (Liu et al., 2020a)	*htd12* (EMS), *t20* (Co-60 radiation; Os12g21710; Rice) (Liu et al., 2020a; Zhou et al., 2021) *zic1-1, zic 1–2, ziso-155* (At1g10830) (EMS; Arabidopsis) (Chen et al., 2010; Cazzonelli et al., 2020) *e2803, ZISO* (Solyc12g098710; EMS; Tomato) (Kachanovsky et al., 2012, Gupta et al., 2021)	Pinalate (Orange) (Rodrigo et al., 2019) *pale yellow9* (Maize) (Li et al., 2007)
*ZDS*	*clb5-3, spc1-1, spc 1-2, spc1-3* (At3g04870; Arabidopsis) (Dong et al., 2007; Avendaño-Vázquez et al., 2014) *alb1, vp-wl2, vp9-8113,vp9-99-2226-1, vp9-R* (GRMZM2G454952; Maize) (Chen et al., 2017; Wang et al., 2020b) *phs2-1, phs2-2* (Os07g10490; Rice) (Fang et al., 2008)		*clb5-1* (EMS), *clb 5–2* (fast-neutron; At3g04870; Arabidopsis) (Avendaño-Vázquez et al., 2014) *ale1* (Os07g10490; EMS; Rice) (Fang et al., 2019)	*nd-1 HaZDS* (GenBank accession no *AJ514406*; sunflower) (Conti et al., 2004)
*CRTISO*	*phs3-1, phs3-2* (Os11g36440; Rice) (Fang et al., 2008) *ccr2-3* (At1g06820; Arabidopsis) (Park et al., 2002)	Chinese kale (Sun et al., 2020a) Tomato (Solyc10g081650) (Dahan-Meir et al., 2018; Lakshmi Jayaraj et al., 2021)	*t^mic^* (fast-neutron), *t3002, t4838, t3406, t9776* (EMS; Solyc10g081650; tomato) (Isaacson et al., 2002; Kachanovsky et al., 2012) *ccr2-1* (EMS), *ccr2-5* (At1g06820; fast-neutron; Arabidopsis) (Park et al., 2002) *yofI* (EMS; Genbank accession number JX491496) (Melon) (Galpaz et al., 2013) *zb2, mit3-1, mit3-2, mit 3–3, mit3-4* (EMS; Os11g36440; Rice) (Zhao et al., 2014; Liu et al., 2018)	*phs3-3* (Os11g36440; Rice) (Fang et al., 2008) Orange flowered calendula (Kishimoto and Ohmiya, 2012) *t3183, LA0351, LA3002, LA3128* (Solyc10g081650; tomato) (Isaacson et al., 2002; Yoo et al., 2017) Chinese cabbage (Bra031539) (Su et al., 2015)
*LCYB*	*lcyB-m2.1* (Maize) (Bai et al., 2009) *phs4-1, phs4-2* (Rice) (Fang et al., 2008)	Tomato *LcyB1* (Solyc04g040190), *LcyB2* (Solyc10g079480) (Li et al., 2018b)	melon (EMS; Cucurbita pepo) (Vicente-Dólera et al., 2014) *szl1* (At2g32640; EMS; Arabidopsis) (Li et al., 2009)	*B, B^og^, B^c^* (Solyc06g074240; Tomato) (Ronen et al., 2000) Red papaya cultivars (Devitt et al., 2010) Capsicum (CA05g00080) (Jeong et al., 2019)
*LCYE*		Tomato (Solyc12g008980) (Li et al., 2018b) Banana (Kaur et al., 2020)	Wheat (EMS) *LCYE-A* (Genbank: EU649785), LCYE-B (Genbank: EU649786) (Richaud et al., 2018) *lut2-1, lut2-2* (EMS; At5g57030; Arabidopsis) (Pogson et al., 1996)	*Delta* (Solyc12g008980; Tomato; Ronen et al., 1999)
*CHY/CYP*	*dsm2*, *cyp97a4-1, cyp97a4-2, cyp97a4-3* (Os04g48880; Rice) (Du et al., 2010; Lv et al., 2012) *lut1-4, lut1-3* (At3g53130)*, lut5-1* (At1g31800)*, b1* (At4g25700)*, b2* (At5g52570)*, nox* (Arabidopsis) (Tian et al., 2003, 2004; Kim and DellaPenna, 2006; Dall’Osto et al., 2013)	Tomato CrtR-b2 (Solyc03g007960) (D’Ambrosio et al., 2018)	*wf1-1* (X-ray), *wf1-2* (EMS), *wf1-3* (Solyc03g007960; fast-neutron; Tomato) (Galpaz et al., 2006) *lut1-1, lut1-2,* (At3g53130) *lut5-2* (At1g31800; EMS; Arabidopsis) (Pogson et al., 1996; Kim and DellaPenna, 2006) E172-3 (EMS; Pepper) (Borovsky et al., 2013)	Orange-rooted carrots (Arango et al., 2014) Maize (Yan et al., 2010) Capsicum (CA03g25820) (Jeong et al., 2019)
*ZEP*	*aba2* (*N. plumbaginifolia*) (Marin et al., 1996) *Osaba1* (Rice) (Agrawal et al., 2001) *aba1-7* (At5g67030; Arabidopsis) (Gonzalez-Jorge et al., 2016)	BnaA09.ZEP, BnaC09.ZEP (*B. napus*) (Liu et al., 2020b)	*hp3-1, hp3-2* (EMS; Solyc02g090890; Tomato) (Galpaz et al., 2008) *aba-1, aba-3, aba-4, npq2-1, npq2-2, aba1-6* (EMS; At5g67030; Arabidopsis) (Duckham et al., 1991; Rock and Zeevaart, 1991; Niyogi et al., 1998; Gonzalez-Jorge et al., 2016)	Capsicum (CA02g10990) (Jeong et al., 2019; Lee et al., 2021)
*VDE*			*npq1-1, npq1-2* (EMS; At1g08550; Arabidopsis) (Niyogi et al., 1998)	
*CCS*				Pepper (CA06g22860; Lefebvre et al., 1998; Popovsky and Paran, 2000; Ha et al., 2007; Guzman et al., 2010; Li et al., 2013b; Jeong et al., 2019).
*ABA4/NXS?*	*aba4-1, aba4-2, aba4-3* (At1g67080; Arabidopsis) (North et al., 2007)		*nxd1-1, nxd1-2* (EMS; Solyc12g041880; Tomato) (Neuman et al., 2014)	

### Carotenoid Cleavage – The Apocarotenoids

Carotenoid molecules can be cleaved at different double bonds by distinct dioxygenases divided into two categories, namely, 9-*cis*-epoxycarotenoid dioxygenases (NCEDs) and carotenoid cleavage dioxygenases (CCDs). The NCEDs are related to the abscisic acid (ABA) production from 9-*cis*-epoxycarotenoids, while CCDs have broader substrate specificities. *CCD7/8* are involved in the synthesis of strigolactones, and *CCD1/4* generate many apocarotenoids with diverse functions. NCED cleaves 9-*cis*-violaxanthin and 9-*cis*-neoxanthin to form xanthoxin, the first committed step in ABA biosynthesis. NCEDs in plants are encoded by many genes having differential expression and roles in different tissues. Mutants have been generated in different NCED genes in maize (*vp14*), Arabidopsis (*nced2*, *nced3*, *nced5*, *nced6,* and *nced9*), tomato (*not*), rice (*nced3* and *nced5*), lettuce (*nced4*), and wheat (*nced1*; Burbidge et al., 1999; Iuchi et al., 2001; Lefebvre et al., 2006; Wan and Li, 2006; Frey et al., 2012; Gonzalez-Jorge et al., 2013; Bertier et al., 2018; Huang et al., 2018b, 2019; Schwartz et al., 2018; Zhang et al., 2019b). All the *nced* mutants, irrespective of the gene mutation, display reduced levels of ABA and hypersensitivity to water stress.

Strigolactones that control shoot branching are generated by the action of *CCD7* and *CCD8*, which act sequentially on 9-*cis*-β-carotene as substrate. Several *CCD7/8* mutants have been isolated and characterized in Arabidopsis (*ccd7/max3* and *ccd8/max4*), petunia (*dad1* and *dad3*), tomato and rice (*htd1* and *d10*), all exhibiting dwarf phenotype and excessive shoot branching (Booker et al., 2004; Snowden et al., 2005; Auldridge et al., 2006; Zou et al., 2006; Arite et al., 2007; Drummond et al., 2009; Hasegawa et al., 2018). Likewise, the CRISPR mutants of *CCD7/8* in rice, tomato, tobacco, and grapevine also show a similar phenotype (Yang et al., 2017; Gao et al., 2018; Bari et al., 2019; Ren et al., 2020). *CCD4* in flowering plants is mainly active in chromoplasts, where it determines coloration in petals and fruits by degrading carotenoid pigments. Knockout mutants of *CCD4* in ipomea, chrysanthemum, and brassica caused white petals to turn pale-yellow/yellow, and in peach, they caused a change from white to yellow-fleshed fruits (Adami et al., 2013; Ma et al., 2014; Zhang et al., 2015; Jo et al., 2016; Watanabe et al., 2018; Wen et al., 2020). In Arabidopsis, *ccd4* and *ccd1* mutants increased seed carotenoid levels, mainly in lutein, neoxanthin, and violaxanthin, with a more pronounced effect in *ccd1* mutants (Gonzalez-Jorge et al., 2013). The list of mutants for carotenoid cleavage is presented in Table 3.

**Table 3:** List of mutants identified in carotenoid degradation pathway.

Gene	Insertional mutagenesis	CRISPR	Induced mutagen	Spontaneous/natural mutation
*NCED*	T5004, 129B08, *nced3-2* (At3g14440)*, nced5-2, nced5-3, nced5-4* (At1g30100)*, nced6-1* (At3g24220)*, nced9-1, nced9-2* (At1g78390)*, nced2-3* (At4g18350; Arabidopsis) (Iuchi et al., 2001; Lefebvre et al., 2006; Wan and Li, 2006; Frey et al., 2012; Gonzalez-Jorge et al., 2013) *vp14-2274, vp*14-3250 (maize) (Schwartz et al., 2018)	*nced5-1, nced5-2, nced3-1, nced3-2* (Rice) (Huang et al., 2018b, 2019) *nced4* (lettuce) (Bertier et al., 2018) *TaNced1* (Genbank accession number JQ772528; Wheat) (Zhang et al., 2019a)	*not* (X-ray), *NCED1* (EMS; Solyc07g056570; Burbidge et al., 1999; Gupta et al., 2017)	
*CCD*	*max3-1, max3-2, max3-3,max3-4, max3-5, max3-6, max3-7, max3-8, max3-11* (At2g44990)*, max4-5, max4-6* (At4g32810)*, ccd1-1* (At3g63520)*, ccd4-1* (At4g19170; Arabidopsis) (Booker et al., 2004; Auldridge et al., 2006; Gonzalez-Jorge et al., 2013)	*ccd7* (Rice) (Yang et al., 2017) *ccd8* (Tomato) (Bari et al., 2019) *ntccd8a, ntccd8b* (Tobacco) (Gao et al., 2018) *VvCCD8* (Grapevine) (Ren et al., 2020) *ccd4* (Ipomoea) (Watanabe et al., 2018)	*max3-9* (Arabidopsis; EMS; At2g44990) (Booker et al., 2004) dad3 (CCD7), dad1-1, dad1-2, dad1-3 (CCD8L EMS; Petunia) (Snowden et al., 2005; Drummond et al., 2009) 2,757, 5,291 (CCD8; Solyc08g066650), CCD4 (Tomato; EMS) (Gupta et al., 2017; Hasegawa et al., 2018) ARTI-Yellow Star (Chrysanthemum; gamma; Ccd4a) (Jo et al., 2016)	*htd1* (CCD7)*, d10-1, d10-2* (CCD8b; Os01g0746400; Rice) (Zou et al., 2006; Arite et al., 2007) 2,127 (Bol029878; Brassica, CCD4) (Zhang et al., 2015) Yellow-fleshed peach (Peach, CCD4) (Adami et al., 2013; Ma et al., 2014; Wen et al., 2020)

### Regulation of Carotenoid Biosynthesis – The Or Perspective

The regulation of carotenoid biosynthesis is complex and depends on many different factors, including the type of tissue and developmental and environmental signals (Sun and Li, 2020). Carotenoid biosynthesis is enhanced following plastid differentiation to chromoplasts, which accumulate large amounts of carotenoids. The tomato mutations *HIGH-PIGMENT1* (*hp1*), *HIGH-PIGMENT2* (*hp2*), and *HIGH-PIGMENT3* (*hp3*) increase chromoplast number and size and thus elevate carotenoid concentration in the fruits (Mustilli et al., 1999; Cookson et al., 2003; Levin et al., 2006; Galpaz et al., 2008; Wang et al., 2008). However, this regulation of carotenoids is indirect. An R2R3-MYB transcription factor was implicated in the regulation of carotenoid biosynthesis in *Mimulus lewisii* flowers based on the analysis of the mutation *Reduced Carotenoid Pigmentation 1* (*RCP1*; Sagawa et al., 2016). Since the transcription of all carotenoid genes was decreased in the *rcp1* flowers, and overexpressing RCP1 decreased anthocyanin production, the effects of R2R3-MYB on the carotenoid pathway are likely indirect. Several other transcriptional and post-translational regulators in different plant species have been proposed as potential regulators of the carotenoid biosynthesis pathway. However, lack of mechanistic aspects of these regulators leaves many gaps in the understanding of their mode of action that do not enable to substantiate their direct effects on specific carotenoid genes or enzymes. The gene ORANGE (OR), is an exceptional case. The role of ORANGE protein as a post-translational regulator of carotenoids accumulation has been well established (Chayut et al., 2017; Kim et al., 2018; Welsch et al., 2018; Osorio, 2019). Therefore, we have included the mutations in the *Or* gene in this review. OR was first identified as a dominant spontaneous mutation in *Brassica oleracea* (*BrOr*), having a retrotransposon insertion in the gene of a plastidial DnaJ, a cysteine-rich domain-containing protein, leading to orange color of inflorescence (Li et al., 2001; Lu et al., 2006). Genome edited lines in rice (*OsOr*) displayed carotenoid accumulation in rice callus (Endo et al., 2019). In melon, a natural gain-of-function mutation (“golden SNP”), Or^HIS^, changes green to orange-fleshed melon fruits (Tzuri et al., 2015). An EMS-induced nonsense mutation in the gene reduced beta-carotene levels in melon fruit (Chayut et al., 2017). OR regulates PSY post-translationally, promotes chromoplast biogenesis, and affects plastid number (Zhou et al., 2015; Sun et al., 2020b). The mutations in the Or gene are listed in Table 4.

**Table 4:** List of mutants identified in orange gene.

Gene	Insertional mutagenesis	CRISPR	Induced mutagen	Spontaneous/natural mutation
*Orange*	*or-1* (At5g61670; Arabidopsis) (Sun et al., 2019)	*OsOr* (Os02g0651300; Rice; Endo et al., 2019)	*CmOr* (low-β; MELO3C005449. Melon; EMS; Chayut et al., 2017)	*CmOr* (MELO3C005449; Melon; Tzuri et al., 2015) *BoOr* Brassica; Li et al., 2001, Lu et al., 2006

## Conclusion and Perspectives

This review aims to assemble all known natural and induced mutants available in the carotenoid metabolic pathway in plants in one place to facilitate comparisons between different plants and assist researchers who seek to study this pathway. Since the founding of the field of biochemical genetic eight decades ago (Beadle, 1945), mutations have been a central tool for researchers to discover biosynthetic pathways and study their regulation. This has also been true in the case of carotenoid biosynthesis in plants, where the genetic approach paved the way for discovering genes and enzymes. Additional utility for mutant isolation and characterization has been known for the physiological and developmental studies of carotenoids in plant life, photosynthesis, developmental processes, and responses to environmental conditions. Finally, due to the contribution of carotenoids to health, mutations in their biosynthesis pathway have made a crucial contribution to the genetic breeding of crop varieties with enhanced nutritional value. Until recent years, the availability of mutations in specific genes was solely dependent on random mutagenesis and selection or screening. Most of these mutants were of loss-of-function nature. The development of the CRISPR/Cas genome editing systems and genomic information of plant species opens new avenues for gene-targeted mutagenesis to generate both leaky and null mutants in those genes and mutation where null mutants are not viable. Genome editing technologies are not restricted to generating mutants and can also be used for biofortification of carotenoids in crop plants. In the future, we will soon reach saturated mutagenesis in model plants and other plant species to better understand the genes’ functions and thus help improve crop plants for a better future.

## Author Contributions

PG and JH have contributed equally in preparing the article.

## Funding

PG is supported by PBC postdoctoral fellowship by Council of Higher Education, Israel and The Hebrew University of Jerusalem, Israel. Research in the laboratory of JH is supported by the Israel Science Foundation Grant No. 1930/18.

## Conflict of Interest

The authors declare that the research was conducted in the absence of any commercial or financial relationships that could be construed as a potential conflict of interest.

## Publisher’s Note

All claims expressed in this article are solely those of the authors and do not necessarily represent those of their affiliated organizations, or those of the publisher, the editors and the reviewers. Any product that may be evaluated in this article, or claim that may be made by its manufacturer, is not guaranteed or endorsed by the publisher.

## References

[ref1] Adami M. De Franceschi P. Brandi F. Liverani A. Giovannini D. Rosati C.. (2013). Identifying a carotenoid cleavage dioxygenase (ccd4) gene controlling yellow/white fruit flesh color of peach. Plant Mol. Biol. Report. 31, 1166–1175. doi: 10.1007/s11105-013-0628-6

[ref2] Agrawal G. K. Yamazaki M. Kobayashi M. Hirochika R. Miyao A. Hirochika H. (2001). Screening of the rice viviparous mutants generated by endogenous retrotransposon Tos17 insertion. Tagging of a zeaxanthin epoxidase gene and a novel OsTATC gene. Plant Physiol. 125, 1248–1257. doi: 10.1104/pp.125.3.1248, PMID: 11244106 PMC65605

[ref3] Ali Z. Abul-Faraj A. Li L. Ghosh N. Piatek M. Mahjoub A.. (2015). Efficient virus-mediated genome editing in plants using the CRISPR/Cas9 system. Mol. Plant 8, 1288–1291. doi: 10.1016/j.molp.2015.02.011, PMID: 25749112

[ref4] Ali Z. Eid A. Ali S. Mahfouz M. M. (2018). Pea early-browning virus-mediated genome editing via the CRISPR/Cas9 system in Nicotiana benthamiana and Arabidopsis. Virus Res. 244, 333–337. doi: 10.1016/j.virusres.2017.10.009, PMID: 29051052

[ref5] Araki N. Kusumi K. Masamoto K. Niwa Y. Iba K. (2000). Temperature-sensitive Arabidopsis mutant defective in 1-deoxy-D-xylulose 5-phosphate synthase within the plastid non-mevalonate pathway of isoprenoid biosynthesis. Physiol. Plant. 108, 19–24. doi: 10.1034/j.1399-3054.2000.108001019.x

[ref6] Arango J. Jourdan M. Geoffriau E. Beyer P. Welsch R. (2014). Carotene hydroxylase activity determines the levels of both α-carotene and total carotenoids in orange carrots. Plant Cell 26, 2223–2233. doi: 10.1105/tpc.113.122127, PMID: 24858934 PMC4079379

[ref7] Arite T. Iwata H. Ohshima K. Maekawa M. Nakajima M. Kojima M.. (2007). DWARF10, an RMS1/MAX4/DAD1 ortholog, controls lateral bud outgrowth in rice. Plant J. 51, 1019–1029. doi: 10.1111/j.1365-313X.2007.03210.x, PMID: 17655651

[ref8] Auldridge M. E. Block A. Vogel J. T. Dabney-Smith C. Mila I. Bouzayen M.. (2006). Characterization of three members of the Arabidopsis carotenoid cleavage dioxygenase family demonstrates the divergent roles of this multifunctional enzyme family. Plant J. 45, 982–993. doi: 10.1111/j.1365-313X.2006.02666.x, PMID: 16507088

[ref9] Avendaño-Vázquez A. O. Cordoba E. Llamas E. San Román C. Nisar N. De la Torre S.. (2014). An uncharacterized apocarotenoid-derived signal generated in ζ-carotene desaturase mutants regulates leaf development and the expression of chloroplast and nuclear genes in Arabidopsis. Plant Cell 26, 2524–2537. doi: 10.1105/tpc.114.123349, PMID: 24907342 PMC4114949

[ref10] Awasthi P. Kocábek T. Mishra A. K. Nath V. S. Shrestha A. Matoušek J. (2021). Establishment of CRISPR/Cas9 mediated targeted mutagenesis in hop (Humulus lupulus). Plant Physiol. Biochem. 160, 1–7. doi: 10.1016/j.plaphy.2021.01.006, PMID: 33445042

[ref11] Bai L. Kim E. H. Dellapenna D. Brutnell T. P. (2009). Novel lycopene epsilon cyclase activities in maize revealed through perturbation of carotenoid biosynthesis. Plant J. 59, 588–599. doi: 10.1111/j.1365-313X.2009.03899.x, PMID: 19392686

[ref12] Banakar R. Eggenberger A. L. Lee K. Wright D. A. Murugan K. Zarecor S.. (2019). High-frequency random DNA insertions upon co-delivery of CRISPR-Cas9 ribonucleoprotein and selectable marker plasmid in rice. Sci. Rep. 9, 1–13. doi: 10.1038/s41598-019-55681-y 31882637 PMC6934568

[ref13] Bánfalvi Z. Csákvári E. Villányi V. Kondrák M. (2020). Generation of transgene-free PDS mutants in potato by agrobacterium-mediated transformation. BMC Biotechnol. 20:25. doi: 10.1186/s12896-020-00621-2, PMID: 32398038 PMC7216596

[ref14] Bari V. K. Nassar J. A. Kheredin S. M. Gal-On A. Ron M. Britt A.. (2019). CRISPR/Cas9-mediated mutagenesis of CAROTENOID CLEAVAGE DIOXYGENASE 8 in tomato provides resistance against the parasitic weed Phelipanche aegyptiaca. Sci. Rep. 9, 1–12. doi: 10.1038/s41598-019-47893-z 31391538 PMC6685993

[ref15] Barja M. V. Ezquerro M. Beretta S. Diretto G. Florez-Sarasa I. Feixes E.. (2021). Several geranylgeranyl diphosphate synthase isoforms supply metabolic substrates for carotenoid biosynthesis in tomato. New Phytol. 231, 255–272. doi: 10.1111/nph.17283, PMID: 33590894

[ref16] Beadle G. W. (1945). Biochemical genetics. Chem. Rev. 37, 15–96. doi: 10.1021/cr60116a002, PMID: 34872044

[ref17] Bernard G. Gagneul D. dos Santos H. A. Etienne A. Hilbert J. L. Rambaud C. (2019). Efficient genome editing using CRISPR/Cas9 technology in chicory. Int. J. Mol. Sci. 20:1155. doi: 10.3390/ijms20051155, PMID: 30845784 PMC6429391

[ref18] Bertier L. D. Ron M. Huo H. Bradford K. J. Britt A. B. Michelmore R. W. (2018). High-resolution analysis of the efficiency, heritability, and editing outcomes of CRISPR/Cas9-induced modifications of NCED4 in lettuce (Lactuca sativa). G3 8, 1513–1521. doi: 10.1534/g3.117.300396 29511025 PMC5940144

[ref238] Booker J. Auldridge M. Wills S. McCarty D. Klee H. Leyser O. (2004). MAX3/CCD7 is a carotenoid cleavage dioxygenase required for the synthesis of a novel plant signaling molecule. Curr. Biol. 14, 1232–1238. doi: 10.1016/j.cub.2004.06.061, PMID: 15268852

[ref19] Borovsky Y. Tadmor Y. Bar E. Meir A. Lewinsohn E. Paran I. (2013). Induced mutation in β-CAROTENE HYDROXYLASE results in accumulation of β-carotene and conversion of red to orange color in pepper fruit. Theor. Appl. Genet. 126, 557–565. doi: 10.1007/s00122-012-2001-9, PMID: 23124390

[ref20] Bouvier F. Hugueney P. D’harlingue A. Kuntz M. Camara B. (1994). Xanthophyll biosynthesis in chromoplasts: isolation and molecular cloning of an enzyme catalyzing the conversion of 5, 6-epoxycarotenoid into ketocarotenoid. Plant J. 6, 45–54. doi: 10.1046/j.1365-313X.1994.6010045.x, PMID: 7920703

[ref21] Buckner B. Miguel P. S. Janick-Buckner D. Bennetzent J. L. (1996). The y1 gene of maize codes for phytoene synthase. Genetics 143, 479–488. doi: 10.1093/genetics/143.1.479 8722797 PMC1207279

[ref22] Budziszewski G. J. Lewis S. P. Glover L. W. Reineke J. Jones G. Zieninik L. S.. (2001). Arabidopsis genes essential for seedling viability: isolation of insertional mutants and molecular cloning. Genetics 159, 1765–1778. doi: 10.1093/genetics/159.4.1765, PMID: 11779813 PMC1461917

[ref23] Burbidge A. Grieve T. M. Jackson A. Thompson A. McCarty D. R. Taylor I. B. (1999). Characterization of the ABA-deficient tomato mutant notabilis and its relationship with maize Vp14. Plant J. 17, 427–431. doi: 10.1046/j.1365-313X.1999.00386.x, PMID: 10205899

[ref24] Cazzonelli C. I. Hou X. Alagoz Y. Rivers J. Dhami N. Lee J.. (2020). A cis-carotene derived apocarotenoid regulates etioplast and chloroplast development. elife 9, 1–32. doi: 10.7554/eLife.45310 PMC6994220 32003746

[ref25] Chamovitz D. Pecker I. Sandmann G. Boger P. Hirschberg J. (1990). Cloning a gene coding for norflurazon resistance in cyanobacteria. Zeitschrift fár Naturforschung 45c, 482–486. 10.1515/znc-1990-0531 2116131

[ref26] Chamovitz D. Sandmann G. Hirschberg J. (1993). Molecular and biochemical characterization of herbicide- resistant mutants of cyanobacteria reveals that phytoene desaturation is a rate-limiting step in carotenoid biosynthesis. J. Biol. Chem. 268, 17348–17353. doi: 10.1016/S0021-9258(19)85341-3, PMID: 8349618

[ref27] Chappell J. (1995). Biochemistry and molecular biology of the isoprenoid biosynthetic pathway in plants. Annu. Rev. Plant Physiol. Plant Mol. Biol. 46, 521–547. doi: 10.1146/annurev.pp.46.060195.002513, PMID: 34814027

[ref28] Charrier A. Vergne E. Dousset N. Richer A. Petiteau A. Chevreau E. (2019). Efficient targeted mutagenesis in apple and first time edition of pear using the CRISPR-Cas9 system. Front. Plant Sci. 10:40. doi: 10.3389/fpls.2019.00040 30787936 PMC6373458

[ref29] Chayut N. Yuan H. Ohali S. Meir A. Sa’ar U. Tzuri G.. (2017). Distinct mechanisms of the ORANGE protein in controlling carotenoid flux. Plant Physiol. 173, 376–389. doi: 10.1104/pp.16.01256 27837090 PMC5210724

[ref30] Chen Y. Li J. Fan K. Du Y. Ren Z. Xu J.. (2017). Mutations in the maize zeta-carotene desaturase gene lead to viviparous kernel. PLoS One 12, 1–17. doi: 10.1371/journal.pone.0174270 PMC5365113 28339488

[ref31] Chen L. Li W. Katin-Grazzini L. Ding J. Gu X. Li Y.. (2018a). A method for the production and expedient screening of CRISPR/Cas9-mediated non-transgenic mutant plants. Hortic. Res. 5:13. doi: 10.1038/s41438-018-0023-4 29531752 PMC5834642

[ref32] Chen L. Li W. Li Y. Feng X. Du K. Wang G.. (2019). Identified trans-splicing of YELLOW-FRUITED TOMATO 2 encoding the PHYTOENE SYNTHASE 1 protein alters fruit color by map-based cloning, functional complementation and RACE. Plant Mol. Biol. 100, 647–658. doi: 10.1007/s11103-019-00886-y, PMID: 31154655

[ref33] Chen Y. Li F. Wurtzel E. T. (2010). Isolation and characterization of the Z-ISO gene encoding a missing component of carotenoid biosynthesis in plants. Plant Physiol. 153, 66–79. doi: 10.1104/pp.110.153916, PMID: 20335404 PMC2862425

[ref34] Chen N. Wang P. Li C. Wang Q. Pan J. Xiao F.. (2018b). A single nucleotide mutation of the IspE gene participating in the MEP pathway for isoprenoid biosynthesis causes a green-revertible yellow leaf phenotype in rice. Plant Cell Physiol. 59, 1905–1917. doi: 10.1093/pcp/pcy108 29893915

[ref35] Conti A. Pancaldi S. Fambrini M. Michelotti V. Bonora A. Salvini M.. (2004). A deficiency at the gene coding for ζ;-carotene desaturase characterizes the sunflower non dormant-1 mutant. Plant Cell Physiol. 45, 445–455. doi: 10.1093/pcp/pch052, PMID: 15111719

[ref36] Cookson P. J. Kiano J. W. Shipton C. A. Fraser P. D. Romer S. Schuch W.. (2003). Increases in cell elongation, plastid compartment size and phytoene synthase activity underlie the phenotype of the *high pigment-1* mutant of tomato. Planta 217, 896–903. doi: 10.1007/s00425-003-1065-9, PMID: 12844264

[ref37] Cordoba E. Salmi M. León P. (2009). Unravelling the regulatory mechanisms that modulate the MEP pathway in higher plants. J. Exp. Bot. 60, 2933–2943. doi: 10.1093/jxb/erp190, PMID: 19584121

[ref38] Crowell D. N. Packard C. E. Pierson C. A. Giner J. L. Downes B. P. Narasimha Chary S. (2003). Identification of an allele of CLA1 associated with variegation in Arabidopsis thaliana. Physiol. Plant. 118, 29–37. doi: 10.1034/j.1399-3054.2003.00063.x, PMID: 12702011

[ref39] Cunningham F. X. Gantt E. (1998). Genes and enzymes of carotenoid biosynthesis in plants. Annu. Rev. Plant Biol. 49, 557–583. doi: 10.1146/annurev.arplant.49.1.557, PMID: 15012246

[ref40] D’Ambrosio C. Stigliani A. L. Giorio G. (2018). CRISPR/Cas9 editing of carotenoid genes in tomato. Transgenic Res. 27, 367–378. doi: 10.1007/s11248-018-0079-9, PMID: 29797189

[ref41] Dahan-Meir T. Filler-Hayut S. Melamed-Bessudo C. Bocobza S. Czosnek H. Aharoni A.. (2018). Efficient in planta gene targeting in tomato using geminiviral replicons and the CRISPR/Cas9 system. Plant J. 95, 5–16. doi: 10.1111/tpj.13932, PMID: 29668111

[ref42] Dai X. Yang X. Wang C. Fan Y. Xin S. Hua Y.. (2021). CRISPR/Cas9-mediated genome editing in Hevea brasiliensis. Ind. Crop. Prod. 164:113418. doi: 10.1016/j.indcrop.2021.113418

[ref43] Dall’Osto L. Piques M. Ronzani M. Molesini B. Alboresi A. Cazzaniga S.. (2013). The Arabidopsis nox mutant lacking carotene hydroxylase activity reveals a critical role for xanthophylls in photosystem I biogenesis. Plant Cell 25, 591–608. doi: 10.1105/tpc.112.108621, PMID: 23396829 PMC3608780

[ref44] Dang H. T. Malone J. M. Boutsalis P. Gill G. Preston C. (2018). The mechanism of diflufenican resistance and its inheritance in oriental mustard (*Sisymbrium orientale* L.) from Australia. Pest Manag. Sci. 74, 1279–1285. doi: 10.1002/ps.4858 29330913

[ref45] De Bruyn C. Ruttink T. Eeckhaut T. Jacobs T. De Keyser E. Goossens A.. (2020). Establishment of CRISPR/Cas9 genome editing in Witloof (Cichorium intybus var. foliosum). Front. Genome Ed. 2:604876. doi: 10.3389/fgeed.2020.604876, PMID: 34713228 PMC8525355

[ref46] Devitt L. C. Fanning K. Dietzgen R. G. Holton T. A. (2010). Isolation and functional characterization of a lycopene β-cyclase gene that controls fruit colour of papaya (Carica papaya L.). J. Exp. Bot. 61, 33–39. doi: 10.1093/jxb/erp284, PMID: 19887502 PMC2791114

[ref47] Dong H. Deng Y. Mu J. Lu Q. Wang Y. Xu Y.. (2007). The Arabidopsis spontaneous cell death 1 gene, encoding a ζ-carotene desaturase essential for carotenoid biosynthesis, is involved in chloroplast development, photoprotection and retrograde signalling. Cell Res. 17, 458–470. doi: 10.1038/cr.2007.37, PMID: 17468780

[ref48] Drummond R. S. M. Marcela Martínez-Sánchez N. Janssen B. J. Templeton K. R. Simons J. L. Quinn B. D.. (2009). Petunia hybrida CAROTENOID CLEAVAGE DIOXYGENASE7 is involved in the production of negative and positive branching signals in petunia. Plant Physiol. 151, 1867–1877. doi: 10.1104/pp.109.146720, PMID: 19846541 PMC2785980

[ref49] Du H. Wang N. Cui F. Li X. Xiao J. Xiong L. (2010). Characterization of the β-carotene hydroxylase gene DSM2 conferring drought and oxidative stress resistance by increasing xanthophylls and abscisic acid synthesis in rice. Plant Physiol. 154, 1304–1318. doi: 10.1104/pp.110.163741, PMID: 20852032 PMC2971608

[ref50] Du H. Zeng X. Zhao M. Cui X. Wang Q. Yang H.. (2016). Efficient targeted mutagenesis in soybean by TALENs and CRISPR/Cas9. J. Biotechnol. 217, 90–97. doi: 10.1016/j.jbiotec.2015.11.005, PMID: 26603121

[ref51] Duckham S. C. Linforth R. S. T. Taylor I. B. (1991). Abscisic-acid-deficient mutants at the aba gene locus of Arabidopsis thaliana are impaired in the epoxidation of zeaxanthin. Plant Cell Environ. 14, 601–606. doi: 10.1111/j.1365-3040.1991.tb01531.x

[ref52] Dutt M. Mou Z. Zhang X. Tanwir S. E. Grosser J. W. (2020). Efficient CRISPR/Cas9 genome editing with citrus embryogenic cell cultures. BMC Biotechnol. 20, 1–7. doi: 10.1186/s12896-020-00652-9 33167938 PMC7654154

[ref53] Endo A. Masafumi M. Kaya H. Toki S. (2016). Efficient targeted mutagenesis of rice and tobacco genomes using Cpf1 from Francisella novicida. Sci. Rep. 6, 1–9. doi: 10.1038/srep38169 27905529 PMC5131344

[ref54] Endo A. Saika H. Takemura M. Misawa N. Toki S. (2019). A novel approach to carotenoid accumulation in rice callus by mimicking the cauliflower Orange mutation via genome editing. Rice 12, 1–5. doi: 10.1186/s12284-019-0345-3 31713832 PMC6851270

[ref55] Estevez J. M. Cantero A. Romero C. Kawaide H. Jimenez L. F. Kuzuyama T.. (2000). Analysis of the expression of CLA1, a gene that encodes the 1-deoxyxylulose 5-phosphate synthase of the 2-C-methyl-D-erythritol-4-phosphate pathway in Arabidopsis. Plant Physiol. 124, 95–103. doi: 10.1104/pp.124.1.95, PMID: 10982425 PMC59125

[ref56] Fan D. Liu T. Li C. Jiao B. Li S. Hou Y.. (2015). Efficient CRISPR/Cas9-mediated targeted mutagenesis in Populus in the first generation. Sci. Rep. 5, 1–7. doi: 10.1038/srep12217 PMC4507398 26193631

[ref57] Fang J. Chai C. Qian Q. Li C. Tang J. Sun L.. (2008). Mutations of genes in synthesis of the carotenoid precursors of ABA lead to pre-harvest sprouting and photo-oxidation in rice. Plant J. 54, 177–189. doi: 10.1111/j.1365-313X.2008.03411.x 18208525 PMC2327239

[ref58] Fang Y. Hou L. Zhang X. Pan J. Ren D. Zeng D.. (2019). Disruption of ζ-carotene desaturase protein ALE1 leads to chloroplast developmental defects and seedling lethality. J. Agric. Food Chem. 67, 11607–11615. doi: 10.1021/acs.jafc.9b05051, PMID: 31560536

[ref59] Fray R. G. Grierson D. (1993). Identification and genetic analysis of normal and mutant phytoene synthase genes of tomato by sequencing, complementation and co-suppression. Plant Mol. Biol. 22, 589–602. doi: 10.1007/BF00047400 8343597

[ref60] Frey A. Effroy D. Lefebvre V. Seo M. Perreau F. Berger A.. (2012). Epoxycarotenoid cleavage by NCED5 fine-tunes ABA accumulation and affects seed dormancy and drought tolerance with other NCED family members. Plant J. 70, 501–512. doi: 10.1111/j.1365-313X.2011.04887.x, PMID: 22171989

[ref61] Fu X. Feng C. Wang C. Yin X. Lu P. Grierson D.. (2014). Involvement of multiple phytoene synthase genes in tissue-and cultivar-specific accumulation of carotenoids in loquat. J. Exp. Bot. 65, 4679–4689. doi: 10.1093/jxb/eru257, PMID: 24935622 PMC4115255

[ref62] Gady A. L. F. Vriezen W. H. Van de Wal M. H. B. J. Huang P. Bovy A. G. Visser R. G. F.. (2012). Induced point mutations in the phytoene synthase 1 gene cause differences in carotenoid content during tomato fruit ripening. Mol. Breed. 29, 801–812. doi: 10.1007/s11032-011-9591-9, PMID: 22408384 PMC3285762

[ref63] Galpaz N. Burger Y. Lavee T. Tzuri G. Sherman A. Melamed T.. (2013). Genetic and chemical characterization of an EMS induced mutation in Cucumis melo CRTISO gene. Arch. Biochem. Biophys. 539, 117–125. doi: 10.1016/j.abb.2013.08.006, PMID: 23973661

[ref64] Galpaz N. Ronen G. Khalfa Z. Zamir D. Hirschberg J. (2006). A chromoplast-specific carotenoid biosynthesis pathway is revealed by cloning of the tomato white-flower locus. Plant Cell 18, 1947–1960. doi: 10.1105/tpc.105.039966 16816137 PMC1533990

[ref65] Galpaz N. Wang Q. Menda N. Zamir D. Hirschberg J. (2008). Abscisic acid deficiency in the tomato mutant high-pigment 3 leading to increased plastid number and higher fruit lycopene content. Plant J. 53, 717–730. doi: 10.1111/j.1365-313X.2007.03362.x, PMID: 17988221

[ref66] Gao J. Wang G. Ma S. Xie X. Wu X. Zhang X.. (2015). CRISPR/Cas9-mediated targeted mutagenesis in Nicotiana tabacum. Plant Mol. Biol. 87, 99–110. doi: 10.1007/s11103-014-0263-0, PMID: 25344637

[ref67] Gao J. Zhang T. Xu B. Jia L. Xiao B. Liu H.. (2018). CRISPR/Cas9-mediated mutagenesis of carotenoid cleavage dioxygenase 8 (CCD8) in tobacco affects shoot and root architecture. Int. J. Mol. Sci. 19:1062. doi: 10.3390/ijms19041062, PMID: 29614837 PMC5979566

[ref68] García-Alcázar M. Giménez E. Pineda B. Capel C. García-Sogo B. Sánchez S.. (2017). Albino T-DNA tomato mutant reveals a key function of 1-deoxy-D-xylulose-5-phosphate synthase (DXS1) in plant development and survival. Sci. Rep. 7, 1–12. doi: 10.1038/srep45333 28350010 PMC5368609

[ref69] Gil M. J. Coego A. Mauch-Mani B. Jordá L. Vera P. (2005). The Arabidopsis csb3 mutant reveals a regulatory link between salicylic acid-mediated disease resistance and the methyl-erythritol 4-phosphate pathway. Plant J. 44, 155–166. doi: 10.1111/j.1365-313X.2005.02517.x, PMID: 16167903

[ref70] Gonzalez-Jorge S. Ha S. H. Magallanes-Lundback M. Gilliland L. U. Zhou A. Lipka A. E.. (2013). Carotenoid cleavage dioxygenase4 is a negative regulator of β-carotene content in Arabidopsis seeds. Plant Cell 25, 4812–4826. doi: 10.1105/tpc.113.119677, PMID: 24368792 PMC3903989

[ref71] Gonzalez-Jorge S. Mehrshahi P. Magallanes-Lundback M. Lipka A. E. Angelovici R. Gore M. A.. (2016). ZEAXANTHIN EPOXIDASE activity potentiates carotenoid degradation in maturing seed. Plant Physiol. 171, 1837–1851. doi: 10.1104/pp.16.00604, PMID: 27208224 PMC4936585

[ref72] Guevara-García A. San Román C. Arroyo A. Cortés M. E. De La Gutiérrez-Nava M. L. León P. (2005). Characterization of the Arabidopsis clb6 mutant illustrates the importance of posttranscriptional regulation of the methyl-d-erythritol 4-phosphate pathway. Plant Cell 17, 628–643. doi: 10.1105/tpc.104.028860, PMID: 15659625 PMC548831

[ref73] Gupta P. Reddaiah B. Salava H. Upadhyaya P. Tyagi K. Sarma S.. (2017). Next-generation sequencing (NGS)-based identification of induced mutations in a doubly mutagenized tomato (*Solanum lycopersicum*) population. Plant J. 92, 495–508. doi: 10.1111/tpj.13654, PMID: 28779536

[ref74] Gupta P. Rodriguez-Franco M. Bodanapu R. Sreelakshmi Y. Sharma R. (2021). Phytoene synthase 2 in tomato fruits remains functional and contributes to abscisic acid formation. bioRxiv [Preprint]. doi: 10.1101/2021.05.10.443524, PMID: 35151443

[ref75] Gutiérrez-Nava M. D. L. L. Gillmor C. S. Jiménez L. F. Guevara-García A. Léon P. (2004). Chloroplast biogenesis genes act cell and noncell autonomously in early chloroplast development. Plant Physiol. 135, 471–482. doi: 10.1104/pp.103.036996, PMID: 15133149 PMC429399

[ref76] Guzman I. Hamby S. Romero J. Bosland P. W. O’connell M. A. (2010). Variability of carotenoid biosynthesis in orange colored *Capsicum* spp. Plant Sci. 179, 49–59. doi: 10.1016/j.plantsci.2010.04.014, PMID: 20582146 PMC2889374

[ref77] Ha S. H. Kim J. B. Park J. S. Lee S. W. Cho K. J. (2007). A comparison of the carotenoid accumulation in *Capsicum* varieties that show different ripening colours: deletion of the *capsanthin-capsorubin synthase* gene is not a prerequisite for the formation of a yellow pepper. J. Exp. Bot. 58, 3135–3144. doi: 10.1093/jxb/erm132, PMID: 17728301

[ref78] Hable W. E. Oishi K. K. Schumaker K. S. (1998). Viviparous-5 encodes phytoene desaturase, an enzyme essential for abscisic acid (ABA) accumulation and seed development in maize. Mol. Gen. Genet. 257, 167–176. doi: 10.1007/s004380050636, PMID: 9491075

[ref79] Hasegawa S. Tsutsumi T. Fukushima S. Okabe Y. Saito J. Katayama M.. (2018). Low infection of Phelipanche aegyptiaca in micro-tom mutants deficient in CAROTENOID CLEAVAGE DIOXYGENASE 8. Int. J. Mol. Sci. 19:2645. doi: 10.3390/ijms19092645, PMID: 30200620 PMC6163878

[ref80] Henderson S. W. Henderson S. T. Goetz M. Koltunow A. M. G. (2020). Efficient crispr/cas9-mediated knockout of an endogenous phytoene desaturase gene in t1 progeny of apomictic hieracium enables new strategies for apomixis gene identification. Genes. 11, 1–16. doi: 10.3390/genes11091064 PMC7563859 32927657

[ref81] Hirschberg J. (2001). Carotenoid biosynthesis in flowering plants. Curr. Opin. Plant Biol. 4, 210–218. doi: 10.1016/S1369-5266(00)00163-1, PMID: 11312131

[ref82] Hojo M. Tasaka M. Shikanai T. (2005). Physiological requirements of the nonmevalonate pathway for photo-acclimation in Arabidopsis. Plant Biotechnol. 22, 39–45. doi: 10.5511/plantbiotechnology.22.39

[ref83] Hooghvorst I. López-Cristoffanini C. Nogués S. (2019). Efficient knockout of phytoene desaturase gene using CRISPR/Cas9 in melon. Sci. Rep. 9, 1–7. doi: 10.1038/s41598-019-53710-4 31745156 PMC6863862

[ref84] Howells R. M. Craze M. Bowden S. Wallington E. J. (2018). Efficient generation of stable, heritable gene edits in wheat using CRISPR/Cas9. BMC Plant Biol. 18, 1–11. doi: 10.1186/s12870-018-1433-z 30285624 PMC6171145

[ref85] Hsieh M. H. Chang C. Y. Hsu S. J. Chen J. J. (2008). Chloroplast localization of methylerythritol 4-phosphate pathway enzymes and regulation of mitochondrial genes in ispD and ispE albino mutants in Arabidopsis. Plant Mol. Biol. 66, 663–673. doi: 10.1007/s11103-008-9297-5, PMID: 18236010

[ref86] Hsieh M. H. Goodman H. M. (2005). The Arabidopsis IspH homolog is involved in the plastid nonmevalonate pathway of isoprenoid biosynthesis. Plant Physiol. 138, 641–653. doi: 10.1104/pp.104.058735, PMID: 15863698 PMC1150385

[ref87] Hsieh M. H. Goodman H. M. (2006). Functional evidence for the involvement of Arabidopsis IspF homolog in the nonmevalonate pathway of plastid isoprenoid biosynthesis. Planta 223, 779–784. doi: 10.1007/s00425-005-0140-9, PMID: 16231155

[ref239] Hu C. Deng G. Sun X. Zuo C. Li C. Kuang R.. (2017). Establishment of an efficient CRISPR/Cas9-mediated gene editing system in banana. Sci. Agric. Sin. 50, 1294–1301. doi: 10.3864/j.issn.0578-1752.2017.07.012

[ref88] Huang Y. Guo Y. Liu Y. Zhang F. Wang Z. Wang H.. (2018b). 9-*cis*-Epoxycarotenoid dioxygenase 3 regulates plant growth and enhances multi-abiotic stress tolerance in rice. Front. Plant Sci. 9:162. doi: 10.3389/fpls.2018.00162 29559982 PMC5845534

[ref89] Huang Y. Jiao Y. Xie N. Guo Y. Zhang F. Xiang Z.. (2019). OsNCED5, a 9-cis-epoxycarotenoid dioxygenase gene, regulates salt and water stress tolerance and leaf senescence in rice. Plant Sci. 287:110188. doi: 10.1016/j.plantsci.2019.110188 31481229

[ref90] Huang R. Wang Y. Wang P. Li C. Xiao F. Chen N.. (2018a). A single nucleotide mutation of IspF gene involved in the MEP pathway for isoprenoid biosynthesis causes yellow-green leaf phenotype in rice. Plant Mol. Biol. 96, 5–16. doi: 10.1007/s11103-017-0668-7 29143298

[ref91] Isaacson T. Ohad I. Beyer P. Hirschberg J. (2004). Analysis in vitro of the enzyme CRTISO establishes a poly-*cis* carotenoid biosynthesis pathway in plants. Plant Physiol. 136, 4246–4255. doi: 10.1104/pp.104.052092, PMID: 15557094 PMC535854

[ref92] Isaacson T. Ronen G. Zamir D. Hirschberg J. (2002). Cloning of tangerine from tomato reveals a carotenoid isomerase essential for the production of β-carotene and xanthophylls in plants. Plant Cell 14, 333–342. doi: 10.1105/tpc.010303, PMID: 11884678 PMC152916

[ref93] Ishizaki T. (2016). CRISPR/Cas9 in rice can induce new mutations in later generations, leading to chimerism and unpredicted segregation of the targeted mutation. Mol. Breed. 36, 1–15. doi: 10.1007/s11032-016-0591-7

[ref94] Iuchi S. Kobayashi M. Taji T. Naramoto M. Seki M. Kato T.. (2001). Regulation of drought tolerance by gene manipulation of 9-cis-epoxycarotenoid dioxygenase, a key enzyme in abscisic acid biosynthesis in Arabidopsis. Plant J. 27, 325–333. doi: 10.1046/j.1365-313x.2001.01096.x, PMID: 11532178

[ref95] Jeong H. B. Kang M. Y. Jung A. Han K. Lee J. H. Jo J.. (2019). Single-molecule real-time sequencing reveals diverse allelic variations in carotenoid biosynthetic genes in pepper (Capsicum spp.). Plant Biotechnol. J. 17, 1081–1093. doi: 10.1111/pbi.13039, PMID: 30467964 PMC6523600

[ref96] Jia H. Nian W. (2014). Targeted genome editing of sweet orange using Cas9/sgRNA. PLoS One 9:e93806. doi: 10.1371/journal.pone.0093806, PMID: 24710347 PMC3977896

[ref97] Jia H. Orbović V. Wang N. (2019). CRISPR-LbCas12a-mediated modification of citrus. Plant Biotechnol. J. 17, 1928–1937. doi: 10.1111/pbi.13109, PMID: 30908830 PMC6737016

[ref98] Jia H. Wang N. (2014). Xcc-facilitated agroinfiltration of citrus leaves: a tool for rapid functional analysis of transgenes in citrus leaves. Plant Cell Rep. 33, 1993–2001. doi: 10.1007/s00299-014-1673-9, PMID: 25146436

[ref99] Jia H. Xu J. Orbović V. Zhang Y. Wang N. (2017). Editing citrus genome via SaCas9/sgRNA system. Front. Plant Sci. 8:2135. doi: 10.3389/fpls.2017.02135 29312390 PMC5732962

[ref100] Jo Y. D. Kim Y. S. Ryu J. Choi H. I. Kim S. W. Kang H. S.. (2016). Deletion of carotenoid cleavage dioxygenase 4a (CmCCD4a) and global up-regulation of plastid protein-coding genes in a mutant chrysanthemum cultivar producing yellow petals. Sci. Hortic. 212, 49–59. doi: 10.1016/j.scienta.2016.09.035

[ref101] Jung K. H. Lee J. Dardick C. Seo Y. S. Cao P. Canlas P.. (2008). Identification and functional analysis of light- responsive unique genes and gene family members in rice. PLoS Genet. 4:e1000164. doi: 10.1371/journal.pgen.1000164, PMID: 18725934 PMC2515340

[ref102] Kachanovsky D. E. Filler S. Isaacson T. Hirschberg J. (2012). Epistasis in tomato color mutations involves regulation of phytoene synthase 1 expression by cis-carotenoids. Proc. Natl. Acad. Sci. U. S. A. 109, 19021–19026. doi: 10.1073/pnas.1214808109 23112190 PMC3503155

[ref103] Kaur N. Alok A. Shivani Kaur N. Pandey P. Awasthi P.. (2018). CRISPR/Cas9-mediated efficient editing in phytoene desaturase (PDS) demonstrates precise manipulation in banana cv. Rasthali genome. Funct. Integr. Genomics 18, 89–99. doi: 10.1007/s10142-017-0577-5, PMID: 29188477

[ref104] Kaur N. Alok A. Shivani Kumar P. Kaur N. Awasthi P.. (2020). CRISPR/Cas9 directed editing of lycopene epsilon-cyclase modulates metabolic flux for β-carotene biosynthesis in banana fruit. Metab. Eng. 59, 76–86. doi: 10.1016/j.ymben.2020.01.008, PMID: 32006663

[ref105] Kim O. R. Cho M. C. Kim B. D. Huh J. H. (2010). A splicing mutation in the gene encoding phytoene synthase causes orange coloration in Habanero pepper fruits. Mol. Cells 30, 569–574. doi: 10.1007/s10059-010-0154-4, PMID: 21120629

[ref106] Kim J. DellaPenna D. (2006). Defining the primary route for lutein synthesis in plants: The role of Arabidopsis carotenoid β-ring hydroxylase CYP97A3. Proc. Natl. Acad. Sci. U. S. A. 103, 3474–3479. doi: 10.1073/pnas.0511207103 16492736 PMC1413914

[ref107] Kim H. S. Ji C. Y. Lee C. J. Kim S. E. Park S. C. Kwak S. S. (2018). Orange: a target gene for regulating carotenoid homeostasis and increasing plant tolerance to environmental stress in marginal lands. J. Exp. Bot. 69, 3393–3400. doi: 10.1093/jxb/ery023 29385615

[ref108] Kishimoto S. Ohmiya A. (2012). Carotenoid isomerase is key determinant of petal color of Calendula officinalis. J. Biol. Chem. 287, 276–285. doi: 10.1074/jbc.M111.300301, PMID: 22069331 PMC3249078

[ref109] Kumagai M. H. Donson J. Della-Cioppa G. Harvey D. Hanley K. Grill L. K. (1995). Cytoplasmic inhibition of carotenoid biosynthesis with virus-derived RNA. Proc. Natl. Acad. Sci. U. S. A. 92, 1679–1683. doi: 10.1073/pnas.92.5.1679 7878039 PMC42583

[ref110] Lakshmi Jayaraj K. Thulasidharan N. Antony A. John M. Augustine R. Chakravartty N.. (2021). Targeted editing of tomato carotenoid isomerase reveals the role of 5′ UTR region in gene expression regulation. Plant Cell Rep. 40, 621–635. doi: 10.1007/s00299-020-02659-0, PMID: 33449143

[ref111] Lee S. Y. Jang S. J. Jeong H. B. Lee S. Y. Venkatesh J. Lee J. H.. (2021). A mutation in Zeaxanthin epoxidase contributes to orange coloration and alters carotenoid contents in pepper fruit (Capsicum annuum). Plant J. 106, 1692–1707. doi: 10.1111/tpj.15264, PMID: 33825226

[ref112] Lee M. H. Lee J. Choi S. A. Kim Y. S. Koo O. Choi S. H.. (2020). Efficient genome editing using CRISPR–Cas9 RNP delivery into cabbage protoplasts via electro-transfection. Plant Biotechnol. Rep. 14, 695–702. doi: 10.1007/s11816-020-00645-2

[ref113] Lefebvre V. Kuntz M. Camara B. Palloix A. (1998). The *capsanthin-capsorubin synthase* gene: a candidate gene for the *y* locus controlling the red fruit colour in pepper. Plant Mol. Biol. 36, 785–789. doi: 10.1023/A:1005966313415, PMID: 9526511

[ref114] Lefebvre V. North H. Frey A. Sotta B. Seo M. Okamoto M.. (2006). Functional analysis of Arabidopsis NCED6 and NCED9 genes indicates that ABA synthesized in the endosperm is involved in the induction of seed dormancy. Plant J. 45, 309–319. doi: 10.1111/j.1365-313X.2005.02622.x, PMID: 16412079

[ref115] Levin I. De Vos C. H. R. Tadmor Y. Bovy A. Lieberman M. Oren-Shamir M.. (2006). High pigment tomato mutants - more than just lycopene (a review). Israel J. Plant Sci. 54, 179–190.

[ref116] Li Z. Ahn T. K. Avenson T. J. Ballottari M. Cruz J. A. Kramer D. M.. (2009). Lutein accumulation in the absence of zeaxanthin restores nonphotochemical quenching in the *Arabidopsis thaliana* npq1 mutant. Plant Cell 21, 1798–1812. doi: 10.1105/tpc.109.066571 19549928 PMC2714924

[ref117] Li R. Li R. Li X. Fu D. Zhu B. Tian H.. (2018a). Multiplexed CRISPR/Cas9-mediated metabolic engineering of γ-aminobutyric acid levels in Solanum lycopersicum. Plant Biotechnol. J. 16, 415–427. doi: 10.1111/pbi.12781 28640983 PMC5787826

[ref118] Li F. Murillo C. Wurtzel E. T. (2007). Maize Y9 encodes a product essential for 15-cis-ζ-carotene isomerization. Plant Physiol. 144, 1181–1189. doi: 10.1104/pp.107.098996, PMID: 17434985 PMC1914175

[ref119] Li J. F. Norville J. E. Aach J. McCormack M. Zhang D. Bush J.. (2013a). Multiplex and homologous recombination-mediated genome editing in Arabidopsis and Nicotiana benthamiana using guide RNA and Cas9. Nat. Biotechnol. 31, 688–691. doi: 10.1038/nbt.2654 23929339 PMC4078740

[ref120] Li L. Paolillo D. J. Parthasarathy M. V. DiMuzio E. M. Garvin D. F. (2001). A novel gene mutation that confers abnormal patterns of β-carotene accumulation in cauliflower (Brassica oleracea var. botrytis). Plant J. 26, 59–67. doi: 10.1046/j.1365-313x.2001.01008.x 11359610

[ref121] Li X. Wang Y. Chen S. Tian H. Fu D. Zhu B.. (2018b). Lycopene is enriched in tomato fruit by CRISPR/Cas9-mediated multiplex genome editing. Front. Plant Sci. 9:559. doi: 10.3389/fpls.2018.00559 29755497 PMC5935052

[ref122] Li Z. Wang S. Gui X. L. Chang X. B. Gong Z. H. (2013b). A further analysis of the relationship between yellow ripe-fruit color and the *capsanthin-capsorubin synthase* gene in pepper (*capsicum* sp.) indicated a new mutant variant in *C. annuum* and a tandem repeat structure in promoter region. PLoS One 8:e61996. doi: 10.1371/journal.pone.0061996 23637942 PMC3630222

[ref123] Li X. Zuo X. Li M. Yang X. Zhi J. Sun H.. (2021). Efficient CRISPR/Cas9-mediated genome editing in Rehmannia glutinosa. Plant Cell Rep. 40, 1695–1707. doi: 10.1007/s00299-021-02797-z, PMID: 34086068

[ref124] Lichtenthaler H. K. (1999). The 1-deoxy-D-xylulose-5-phosphate pathway of isoprenoid biosynthesis in plants. Annu. Rev. Plant Biol. 50, 47–65. doi: 10.1146/annurev.arplant.50.1.47, PMID: 15012203

[ref241] Liu G. Li J. Godwin I. D. (2019). “Genome editing by CRISPR/Cas9 in sorghum through biolistic bombardment,” in Sorghum, Methods in Molecular Biology, 1931. New York, NY: Humana Press, 169–183. 10.1007/978-1-4939-9039-9_12 30652290

[ref126] Liu L. Xie T. Peng P. Qiu H. Zhao J. Fang J.. (2018). Mutations in the MIT3 gene encoding a caroteniod isomerase lead to increased tiller number in rice. Plant Sci. 267, 1–10. doi: 10.1016/j.plantsci.2017.11.001, PMID: 29362087

[ref240] Liu T. Fan D. Ran L. Jiang Y. Liu R. Luo K. (2015). Highly efficient CRISPR/Cas9-mediated targeted mutagenesis of multiple genes in Populus. Yi Chuan 37, 1044–1052. doi: 10.16288/j.yczz.15-303, PMID: 26496757

[ref125] Liu X. Hu Q. Yan J. Sun K. Liang Y. Jia M.. (2020a). ζ-carotene isomerase suppresses tillering in rice through the coordinated biosynthesis of strigolactone and abscisic acid. Mol. Plant 13, 1784–1801. doi: 10.1016/j.molp.2020.10.001 33038484

[ref127] Liu Y. Ye S. Yuan G. Ma X. Heng S. Yi B.. (2020b). Gene silencing of BnaA09.ZEP and BnaC09.ZEP confers orange color in Brassica napus flowers. Plant J. 104, 932–949. doi: 10.1111/tpj.14970 32808386

[ref128] Lu X. M. Hu X. J. Zhao Y. Z. Song W. B. Zhang M. Chen Z. L.. (2012). Map-based cloning of zb7 encoding an IPP and DMAPP synthase in the MEP pathway of maize. Mol. Plant 5, 1100–1112. doi: 10.1093/mp/sss038, PMID: 22498772

[ref129] Lu S. Van Eck J. Zhou X. Lopez A. B. O’Halloran D. M. Cosman K. M.. (2006). The cauliflower Or gene encodes a DnaJ cysteine-rich domain-containing protein that mediates high levels of β-carotene accumulation. Plant Cell 18, 3594–3605. doi: 10.1105/tpc.106.046417 17172359 PMC1785402

[ref130] Lv M. Z. Chao D. Y. Shan J. X. Zhu M. Z. Shi M. Gao J. P.. (2012). Rice carotenoid β-ring hydroxylase CYP97A4 is involved in lutein biosynthesis. Plant Cell Physiol. 53, 987–1002. doi: 10.1093/pcp/pcs041, PMID: 22470056

[ref131] Ma J. Li J. Zhao J. Zhou H. Ren F. Wang L.. (2014). Inactivation of a gene encoding carotenoid cleavage dioxygenase (CCD4) leads to carotenoid-based yellow coloration of fruit flesh and leaf midvein in peach. Plant Mol. Biol. Report. 32, 246–257. doi: 10.1007/s11105-013-0650-8

[ref132] Ma C. Liu M. Li Q. Si J. Ren X. Song H. (2019a). Efficient BoPDS gene editing in cabbage by the CRISPR/Cas9 system. Hortic. Plant J. 5, 164–169. doi: 10.1016/j.hpj.2019.04.001

[ref133] Ma C. Zhu C. Zheng M. Liu M. Zhang D. Liu B.. (2019b). CRISPR/Cas9-mediated multiple gene editing in Brassica oleracea var. capitata using the endogenous tRNA-processing system. Hortic. Res. 6, 1–15. doi: 10.1038/s41438-018-0107-1 30729010 PMC6355899

[ref134] Mandel M. A. Feldmann K. A. Herrera-Estrella L. Rocha-Sosa M. León P. (1996). CLA1, a novel gene required for chloroplast development, is highly conserved in evolution. Plant J. 9, 649–658. doi: 10.1046/j.1365-313X.1996.9050649.x 8653115

[ref135] Maoka T. (2020). Carotenoids as natural functional pigments. J. Nat. Med. 74, 1–16. doi: 10.1007/s11418-019-01364-x, PMID: 31588965 PMC6949322

[ref136] Marin E. Nussaume L. Quesada A. Gonneau M. Sotta B. Hugueney P.. (1996). Molecular identification of zeaxanthin epoxidase of Nicotiana plumbaginifolia, a gene involved in abscisic acid biosynthesis and corresponding to the ABA locus of Arabidopsis thaliana. EMBO J. 15, 2331–2342. doi: 10.1002/j.1460-2075.1996.tb00589.x, PMID: 8665840 PMC450162

[ref242] Meléndez-Martínez A. J. Böhm V. Borge G. I. A. Cano M. P. Fikselová M. Gruskiene R.. (2021). Carotenoids: considerations for their use in functional foods, nutraceuticals, nutricosmetics, supplements, botanicals, and novel foods in the context of sustainability, circular economy, and climate change. Annu. Rev. Food. 12, 433–460. doi: 10.1146/annurev-food-062220-013218, PMID: 33467905

[ref137] Meng Y. Hou Y. Wang H. Ji R. Liu B. Wen J.. (2017). Targeted mutagenesis by CRISPR/Cas9 system in the model legume Medicago truncatula. Plant Cell Rep. 36, 371–374. doi: 10.1007/s00299-016-2069-9, PMID: 27834007

[ref138] Michel A. Arias R. S. Scheffler B. E. Duke S. O. Netherland M. Dayan F. E. (2004). Somatic mutation-mediated evolution of herbicide resistance in the nonindigenous invasive plant hydrilla (*Hydrilla verticillata*). Mol. Ecol. 13, 3229–3237. doi: 10.1111/j.1365-294X.2004.02280.x, PMID: 15367135

[ref139] Murovec J. Guček K. Bohanec B. Avbelj M. Jerala R. (2018). DNA-free genome editing of brassica oleracea and B. Rapa protoplasts using CRISPR-cas9 ribonucleoprotein complexes. Front. Plant Sci. 9:1594. doi: 10.3389/fpls.2018.01594 30455712 PMC6230560

[ref140] Mustilli A. C. Fenzi F. Ciliento R. Alfano F. Bowler C. (1999). Phenotype of the tomato *high pigment-2* mutant is caused by a mutation in the tomato homolog of *DEETIOLATED1*. Plant Cell 11, 145–157. doi: 10.1105/tpc.11.2.145 9927635 PMC144164

[ref141] Naim F. Dugdale B. Kleidon J. Brinin A. Shand K. Waterhouse P.. (2018). Gene editing the phytoene desaturase alleles of Cavendish banana using CRISPR/Cas9. Transgenic Res. 27, 451–460. doi: 10.1007/s11248-018-0083-0, PMID: 29987710 PMC6156769

[ref142] Nakajima I. Ban Y. Azuma A. Onoue N. Moriguchi T. Yamamoto T.. (2017). CRISPR/Cas9-mediated targeted mutagenesis in grape. PLoS One 12, 1–16. doi: 10.1371/journal.pone.0177966 PMC5436839 28542349

[ref243] Nekrasov V. Staskawicz B. Weigel D. Jones J. D. Kamoun S. (2013). Targeted mutagenesis in the model plant *Nicotiana benthamiana* using Cas9 RNA-guided endonuclease. Nat. Biotechnol. 31, 691–693. doi: 10.1038/nbt.2655, PMID: 23929340

[ref143] Neuman H. Galpaz N. Cunningham F. X. Jr. Zamir D. Hirschberg J. (2014). The tomato mutation nxd1 reveals a gene necessary for neoxanthin biosynthesis and demonstrates that violaxanthin is a sufficient precursor for abscisic acid biosynthesis. Plant J. 78, 80–93. doi: 10.1111/tpj.12451, PMID: 24506237

[ref144] Nisar N. Li L. Lu S. Khin N. C. Pogson B. J. (2015). Carotenoid metabolism in plants. Mol. Plant 8, 68–82. doi: 10.1016/j.molp.2014.12.007, PMID: 25578273

[ref145] Nishitani C. Hirai N. Komori S. Wada M. Okada K. Osakabe K.. (2016). Efficient genome editing in apple using a CRISPR/Cas9 system. Sci. Rep. 6, 1–8. doi: 10.1038/srep31481 27530958 PMC4987624

[ref146] Niyogi K. K. Grossman A. R. Björkman O. (1998). Arabidopsis mutants define a central role for the xanthophyll cycle in the regulation of photosynthetic energy conversion. Plant Cell 10, 1121–1134. doi: 10.1105/tpc.10.7.1121, PMID: 9668132 PMC144052

[ref147] North H. M. Almeida A. D. Boutin J. P. Frey A. To A. Botran L.. (2007). The Arabidopsis ABA-deficient mutant aba4 demonstrates that the major route for stress-induced ABA accumulation is via neoxanthin isomers. Plant J. 50, 810–824. doi: 10.1111/j.1365-313X.2007.03094.x, PMID: 17470058

[ref148] Ntui V. O. Tripathi J. N. Tripathi L. (2020). Robust CRISPR/Cas9 mediated genome editing tool for banana and plantain (Musa spp.). Curr. Plant Biol. 21:100128. doi: 10.1016/j.cpb.2019.100128

[ref149] Odipio J. Alicai T. Ingelbrecht I. Nusinow D. A. Bart R. Taylor N. J. (2017). Efficient CRISPR/cas9 genome editing of phytoene desaturase in cassava. Front. Plant Sci. 8:1780. doi: 10.3389/fpls.2017.01780 29093724 PMC5651273

[ref150] Okada K. Kasahara H. Yamaguchi S. Kawaide H. Kamiya Y. Nojiri H.. (2008). Genetic evidence for the role of isopentenyl diphosphate isomerases in the mevalonate pathway and plant development in Arabidopsis. Plant Cell Physiol. 49, 604–616. doi: 10.1093/pcp/pcn032, PMID: 18303110

[ref151] Oleszkiewicz T. Klimek-Chodacka M. Kruczek M. Godel-Jędrychowska K. Sala K. Milewska-Hendel A.. (2021). Inhibition of carotenoid biosynthesis by crispr/cas9triggers cell wall remodelling in carrot. Int. J. Mol. Sci. 22:6516. doi: 10.3390/ijms22126516, PMID: 34204559 PMC8234013

[ref152] Osakabe Y. Liang Z. Ren C. Nishitani C. Osakabe K. Wada M.. (2018). CRISPR–Cas9-mediated genome editing in apple and grapevine. Nat. Protoc. 13, 2844–2863. doi: 10.1038/s41596-018-0067-9, PMID: 30390050

[ref153] Osorio C. E. (2019). The role of orange gene in carotenoid accumulation: manipulating chromoplasts toward a colored future. Front. Plant Sci. 10:1235. doi: 10.3389/fpls.2019.01235 31636649 PMC6788462

[ref154] Pan C. Ye L. Qin L. Liu X. He Y. Wang J.. (2016). CRISPR/Cas9-mediated efficient and heritable targeted mutagenesis in tomato plants in the first and later generations. Sci. Rep. 6, 2–10. doi: 10.1038/srep24765 27097775 PMC4838866

[ref155] Pankratov I. McQuinn R. Schwartz J. Bar E. Fei Z. Lewinsohn E.. (2016). Fruit carotenoid-deficient mutants in tomato reveal a function of the plastidial isopentenyl diphosphate isomerase (IDI1) in carotenoid biosynthesis. Plant J. 88, 82–94. doi: 10.1111/tpj.13232, PMID: 27288653

[ref156] Park H. Kreunen S. S. Cuttriss A. J. DellaPenna D. Pogson B. J. (2002). Identification of the carotenoid isomerase provides insight into carotenoid biosynthesis, prolamellar body formation, and photomorphogenesis. Plant Cell 14, 321–332. doi: 10.1105/tpc.010302, PMID: 11884677 PMC152915

[ref157] Perreau F. Frey A. Effroy-Cuzzi D. Savane P. Berger A. Gissot L.. (2020). ABSCISIC ACID-DEFICIENT4 has an essential function in both cis-violaxanthin and cis-neoxanthin synthesis. Plant Physiol. 184, 1303–1316. doi: 10.1104/pp.20.00947, PMID: 32883757 PMC7608147

[ref158] Phillips M. A. D’Auria J. C. Gershenzon J. Pichersky E. (2008a). The *Arabidopsis thaliana* type I isopentenyl diphosphate isomerases are targeted to multiple subcellular compartments and have overlapping functions in isoprenoid biosynthesis. Plant Cell 20, 677–696. doi: 10.1105/tpc.107.053926 18319397 PMC2329938

[ref159] Phillips M. A. León P. Boronat A. Rodríguez-Concepción M. (2008b). The plastidial MEP pathway: unified nomenclature and resources. Trends Plant Sci. 13, 619–623. doi: 10.1016/j.tplants.2008.09.003 18948055

[ref160] Pogson B. McDonald K. A. Truong M. Britton G. DellaPenna D. (1996). Arabidopsis carotenoid mutants demonstrate that lutein is not essential for photosynthesis in higher plants. Plant Cell 8, 1627–1639. doi: 10.1105/tpc.8.9.1627, PMID: 8837513 PMC161303

[ref161] Pollack A. J. Gong X. Pollack J. R. (2019). A common phytoene synthase mutation underlies white petal varieties of the California poppy. Sci. Rep. 9, 1–7. doi: 10.1038/s41598-019-48122-3 31406151 PMC6690985

[ref162] Popovsky S. Paran I. (2000). Molecular genetics of the *y* locus in pepper: its relation to *capsanthin-capsorubin synthase* and to fruit color. Theor. Appl. Genet. 101, 86–89. doi: 10.1007/s001220051453

[ref163] Qin G. Gu H. Ma L. Peng Y. Deng X. W. Chen Z.. (2007). Disruption of phytoene desaturase gene results in albino and dwarf phenotypes in Arabidopsis by impairing chlorophyll, carotenoid, and gibberellin biosynthesis. Cell Res. 17, 471–482. doi: 10.1038/cr.2007.40, PMID: 17486124

[ref164] Rao A. V. Rao L. G. (2007). Carotenoids and human health. Pharmacol. Res. 55, 207–216. doi: 10.1016/j.phrs.2007.01.012, PMID: 17349800

[ref165] Ratcliff F. Martin-Hernandez A. M. Baulcombe D. C. (2001). Tobacco rattle virus as a vector for analysis of gene function by silencing. Plant J. 25, 237–245. doi: 10.1046/j.0960-7412.2000.00942.x, PMID: 11169199

[ref166] Ren C. Guo Y. Gathunga E. K. Duan W. Li S. Liang Z. (2019a). Recovery of the non-functional EGFP-assisted identification of mutants generated by CRISPR/Cas9. Plant Cell Rep. 38, 1541–1549. doi: 10.1007/s00299-019-02465-3 31446470

[ref167] Ren C. Guo Y. Kong J. Lecourieux F. Dai Z. Li S.. (2020). Knockout of VvCCD8 gene in grapevine affects shoot branching. BMC Plant Biol. 20, 1–8. doi: 10.1186/s12870-020-2263-3 31996144 PMC6990564

[ref168] Ren C. Liu Y. Guo Y. Duan W. Fan P. Li S.. (2021). Optimizing the CRISPR/Cas9 system for genome editing in grape by using grape promoters. Hortic. Res. 8:52. doi: 10.1038/s41438-021-00489-z, PMID: 33642575 PMC7917103

[ref169] Ren F. Ren C. Zhang Z. Duan W. Lecourieux D. Li S.. (2019b). Efficiency optimization of CRISPR/CAS9-mediated targeted mutagenesis in grape. Front. Plant Sci. 10:612. doi: 10.3389/fpls.2019.00612 31156675 PMC6532431

[ref170] Richaud D. Stange C. Gadaleta A. Colasuonno P. Parada R. Schwember A. R. (2018). Identification of lycopene epsilon cyclase (LCYE) gene mutants to potentially increase β-carotene content in durum wheat (Triticum turgidum L.ssp. Durum) through TILLING. PLoS One 13, 1–17. doi: 10.1371/journal.pone.0208948 PMC6287857 30532162

[ref244] Robertson D. S. Anderson I. C. (1961). Temperature-sensitive alleles of the Y1 locus in maize. J. Hered. 52, 53–60. doi: 10.1093/oxfordjournals.jhered.a107024

[ref171] Rock C. D. Zeevaart J. A. D. (1991). The aba mutant of *Arabidopsis thaliana* is impaired in epoxy-carotenoid biosynthesis. Proc. Natl. Acad. Sci. U. S. A. 88, 7496–7499. doi: 10.1073/pnas.88.17.7496 11607209 PMC52327

[ref172] Rodrigo M. J. Lado J. Alós E. Alquézar B. Dery O. Hirschberg J.. (2019). A mutant allele of ζ-carotene isomerase (Z-ISO) is associated with the yellow pigmentation of the “pinalate” sweet orange mutant and reveals new insights into its role in fruit carotenogenesis. BMC Plant Biol. 19, 1–16. doi: 10.1186/s12870-019-2078-2 31684878 PMC6829850

[ref245] Rodríguez-Concepción M. Boronat A. (2002). Elucidation of the methylerythritol phosphate pathway for isoprenoid biosynthesis in bacteria and plastids. A metabolic milestone achieved through genomics. Plant Physiol. 130, 1079–1089. doi: 10.1104/pp.007138, PMID: 12427975 PMC1540259

[ref173] Rohdich F. Lauw S. Kaiser J. Feicht R. Kohler P. Bacher A.. (2006). Isoprenoid biosynthesis in plants – 2C-methyl-D-erythritol-4-phosphate synthase (IspC protein) of *Arabidopsis thaliana*. FEBS J. 273, 4446–4458. doi: 10.1111/j.1742-4658.2006.05446.x, PMID: 16972937

[ref174] Ronen G. Carmel-Goren L. Zamir D. Hirschberg J. (2000). An alternative pathway to β-carotene formation in plant chromoplasts discovered by map-based cloning of Beta and old-gold color mutations in tomato. Proc. Natl. Acad. Sci. U. S. A. 97, 11102–11107. doi: 10.1073/pnas.190177497 10995464 PMC27155

[ref175] Ronen G. Cohen M. Zamir D. Hirschberg J. (1999). Regulation of carotenoid biosynthesis during tomato fruit development: expression of the gene for lycopene epsilon-cyclase is down-regulated during ripening and is elevated in the mutant *Delta*. Plant J. 17, 341–351. doi: 10.1046/j.1365-313X.1999.00381.x, PMID: 10205893

[ref176] Ruiz-Sola M. Á. Coman D. Beck G. Barja M. V. Colinas M. Graf A.. (2016). Arabidopsis GERANYLGERANYL DIPHOSPHATE SYNTHASE 11 is a hub isozyme required for the production of most photosynthesis-related isoprenoids. New Phytol. 209, 252–264. doi: 10.1111/nph.13580, PMID: 26224411

[ref177] Ruiz-Sola M. Á. Rodríguez-Concepción M. (2012). Carotenoid biosynthesis in Arabidopsis: A colorful pathway. Arabidopsis Book 10:e0158. doi: 10.1199/tab.0158 22582030 PMC3350171

[ref178] Ruppel N. J. Kropp K. N. Davis P. A. Martin A. E. Luesse D. R. Hangarter R. P. (2013). Mutations in geranylgeranyl diphosphate synthase 1 affect chloroplast development in *Arabidopsis thaliana* (Brassicaceae). Am. J. Bot. 100, 2074–2084. doi: 10.3732/ajb.1300124, PMID: 24081146

[ref179] Sagawa J. M. Stanley L. E. LaFountain A. M. Frank H. A. Liu C. Yuan Y. W. (2016). An R2R3-MYB transcription factor regulates carotenoid pigmentation in *Mimulus lewisii* flowers. New Phytol. 209, 1049–1057. doi: 10.1111/nph.13647 26377817

[ref180] Schmitz D. J. Ali Z. Wang C. Aljedaani F. Hooykaas P. J. J. Mahfouz M.. (2020). CRISPR/Cas9 mutagenesis by translocation of Cas9 protein into plant cells via the agrobacterium type IV secretion system. Front. Genome Ed. 2:6. doi: 10.3389/fgeed.2020.00006 34713215 PMC8525350

[ref181] Schwartz S. H. Tan B. C. Gage D. A. Zeevaart J. A. D. Schwartz S. H. Tan B. C.. (2018). Specific oxidative cleavage of carotenoids by VP14 of maize. Science 276, 1872–1874. doi: 10.1126/science.276.5320.1872 9188535

[ref182] Shan S. Mavrodiev E. V. Li R. Zhang Z. Hauser B. A. Soltis P. S.. (2018). Application of CRISPR/Cas9 to Tragopogon (Asteraceae), an evolutionary model for the study of polyploidy. Mol. Ecol. Resour. 18, 1427–1443. doi: 10.1111/1755-0998.12935, PMID: 30086204

[ref246] Shan Q. Wang Y. Li J. Zhang Y. Chen K. Liang Z.. (2013). Targeted genome modification of crop plants using a CRISPR-Cas system. Nat. Biotechnol. 31, 686–688. doi: 10.1038/nbt.2650, PMID: 23929338

[ref183] Snowden K. C. Simkin A. J. Janssen B. J. Templeton K. R. Loucas H. M. Simons J. L.. (2005). The decreased apical dominance1/Petunia hybrida carotenoid cleavage dioxygenase8 gene affects branch production and plays a role in leaf senescence, root growth, and flower development. Plant Cell 17, 746–759. doi: 10.1105/tpc.104.027714, PMID: 15705953 PMC1069696

[ref184] Su T. Yu S. Zhang J. W. F. Yu Y. Zhang D. Zhao X.. (2015). Loss of function of the carotenoid isomerase gene BrCRTISO confers orange color to the inner leaves of Chinese cabbage (Brassica rapa L. ssp. pekinensis). Plant Mol. Biol. Report. 33, 648–659. doi: 10.1007/s11105-014-0779-0

[ref185] Suarez J. V. Banks S. Thomas P. G. Day A. (2014). A new F131V mutation in Chlamydomonas phytoene desaturase locates a cluster of norflurazon resistance mutations near the FAD-binding site in 3D protein models. PLoS ONE. 9:e99894. doi: 10.1371/journal.pone.0099894, PMID: 24936791 PMC4061028

[ref186] Sun B. Jiang M. Zheng H. Jian Y. Huang W. L. Yuan Q.. (2020a). Color-related chlorophyll and carotenoid concentrations of Chinese kale can be altered through CRISPR/Cas9 targeted editing of the carotenoid isomerase gene BoaCRTISO. Hortic. Res. 7, 1–11. doi: 10.1038/s41438-020-00379-w, PMID: 33082968 PMC7527958

[ref187] Sun T. Li L. (2020). Toward the ‘golden’ era: the status in uncovering the regulatory control of carotenoid accumulation in plants. Plant Sci. 290:110331. doi: 10.1016/j.plantsci.2019.110331, PMID: 31779888

[ref188] Sun T. Yuan H. Chen C. Kadirjan-Kalbach D. K. Mazourek M. Osteryoung K. W.. (2020b). OR^His^, a natural variant of OR, specifically interacts with plastid division factor ARC3 to regulate chromoplast number and carotenoid accumulation. Mol. Plant 13, 864–878. doi: 10.1016/j.molp.2020.03.007, PMID: 32222485

[ref189] Sun B. Zheng A. Jiang M. Xue S. Yuan Q. Jiang L.. (2018). CRISPR/Cas9-mediated mutagenesis of homologous genes in Chinese kale. Sci. Rep. 8, 1–10. doi: 10.1038/s41598-018-34884-9 30429497 PMC6235979

[ref190] Sun T. Zhou F. Huang X. Q. Chen W. C. Kong M. J. Zhou C. F.. (2019). ORANGE represses chloroplast biogenesis in etiolated Arabidopsis cotyledons via interaction with TCP14. Plant Cell 31, 2996–3014. doi: 10.1105/tpc.18.00290 31604812 PMC6925005

[ref191] Syombua E. D. Zhang Z. Tripathi J. N. Ntui V. O. Kang M. George O. O.. (2021). A CRISPR/Cas9-based genome-editing system for yam (Dioscorea spp.). Plant Biotechnol. J. 19, 645–647. doi: 10.1111/pbi.13515, PMID: 33222361 PMC8051594

[ref192] Taparia Y. Zarka A. Leu S. Zarivach R. Boussiba S. Khozin-Goldberg I. (2019). A novel endogenous selection marker for the diatom *Phaeodactylum tricornutum* based on a unique mutation in phytoene desaturase 1. Sci. Rep. 9, 8217–44710. doi: 10.1038/s41598-019-44710-5 31160749 PMC6546710

[ref193] Tian S. Jiang L. Gao Q. Zhang J. Zong M. Zhang H.. (2017). Efficient CRISPR/Cas9-based gene knockout in watermelon. Plant Cell Rep. 36, 399–406. doi: 10.1007/s00299-016-2089-5, PMID: 27995308

[ref194] Tian L. Magallanes-Lundback M. Musetti V. DellaPenna D. (2003). Functional analysis of β- and ε-ring carotenoid hydroxylases in Arabidopsis. Plant Cell 15, 1320–1332. doi: 10.1105/tpc.011403, PMID: 12782726 PMC156369

[ref195] Tian L. Musetti V. Kim J. Magallanes-Lundback M. DellaPenna D. (2004). The Arabidopsis LUT1 locus encodes a member of the cytochrome P450 family that is required for carotenoid ε-ring hydroxylation activity. Proc. Natl. Acad. Sci. U. S. A. 101, 402–407. doi: 10.1073/pnas.2237237100 14709673 PMC314197

[ref196] Tritsch D. Hemmerlin A. Bach T. J. Rohmer M. (2010). Plant isoprenoid biosynthesis via the MEP pathway: in vivo IPP/DMAPP ratio produced by (E)-4-hydroxy-3-methylbut-2-enyl diphosphate reductase in tobacco BY-2 cell cultures. FEBS Lett. 584, 129–134. doi: 10.1016/j.febslet.2009.11.010, PMID: 19903472

[ref197] Tsutsui H. Higashiyama T. (2017). PKAMA-ITACHI vectors for highly efficient CRISPR/Cas9-mediated gene knockout in *Arabidopsis thaliana*. Plant Cell Physiol. 58, 46–56. doi: 10.1093/pcp/pcw191, PMID: 27856772 PMC5444565

[ref198] Tzuri G. Zhou X. Chayut N. Yuan H. Portnoy V. Meir A.. (2015). A ‘golden’SNP in CmOr governs the fruit flesh color of melon (Cucumis melo). Plant J. 82, 267–279. doi: 10.1111/tpj.12814 25754094

[ref199] Upadhyay S. K. Kumar J. Alok A. Tuli R. (2013). RNA-guided genome editing for target gene mutations in wheat. G3 3, 2233–2238. doi: 10.1534/g3.113.008847 24122057 PMC3852385

[ref200] Vicente-Dólera N. Troadec C. Moya M. Del Río-Celestino M. Pomares-Viciana T. Bendahmane A.. (2014). First TILLING platform in cucurbita pepo: A new mutant resource for gene function and crop improvement. PLoS One 9:e112743. doi: 10.1371/journal.pone.0112743, PMID: 25386735 PMC4227871

[ref201] Wagner T. Windhovel U. Romer S. (2002). Transformation of tobacco with a mutated cyanobacterial phytoene desaturase gene confers resistance to bleaching herbicides. Zeitschrift fur Naturforschung C 57, 671–679. doi: 10.1515/znc-2002-7-821, PMID: 12240995

[ref202] Wan X. R. Li L. (2006). Regulation of ABA level and water-stress tolerance of Arabidopsis by ectopic expression of a peanut 9-cis-epoxycarotenoid dioxygenase gene. Biochem. Biophys. Res. Commun. 347, 1030–1038. doi: 10.1016/j.bbrc.2006.07.026, PMID: 16870153

[ref203] Wang J. Y. Lin P. Y. Al-Babili S. (2020c). On the biosynthesis and evolution of apocarotenoid plant growth regulators. Semin. Cell Dev. Biol. 10, 3–11. doi: 10.1016/j.semcdb.2020.07.007 32732130

[ref204] Wang S. Liu J. Feng Y. Niu X. Giovannoni J. Liu Y. (2008). Altered plastid levels and potential for improved fruit nutrient content by downregulation of the tomato DDB1-interacting protein CUL4. Plant J. 55, 89–103. doi: 10.1111/j.1365-313X.2008.03489.x, PMID: 18363785

[ref205] Wang Z. Wang S. Li D. Zhang Q. Li L. Zhong C.. (2018). Optimized paired-sgRNA/Cas9 cloning and expression cassette triggers high-efficiency multiplex genome editing in kiwifruit. Plant Biotechnol. J. 16, 1424–1433. doi: 10.1111/pbi.12884, PMID: 29331077 PMC6041439

[ref206] Wang J. Wu H. Chen Y. Yin T. (2020a). Efficient CRISPR/Cas9-mediated gene editing in an interspecific hybrid poplar with a highly heterozygous genome. Front. Plant Sci. 11:996. doi: 10.3389/fpls.2020.00996 32719704 PMC7347981

[ref207] Wang S. Zhang S. Wang W. Xiong X. Meng F. Cui X. (2015). Efficient targeted mutagenesis in potato by the CRISPR/Cas9 system. Plant Cell Rep. 34, 1473–1476. doi: 10.1007/s00299-015-1816-7, PMID: 26082432

[ref208] Wang M. Zhu X. Li Y. Xia Z. (2020b). Transcriptome analysis of a new maize albino mutant reveals that zeta-carotene desaturase is involved in chloroplast development and retrograde signaling. Plant Physiol. Biochem. 156, 407–419. doi: 10.1016/j.plaphy.2020.09.025 33010551

[ref209] Watanabe K. Oda-Yamamizo C. Sage-Ono K. Ohmiya A. Ono M. (2018). Alteration of flower colour in Ipomoea nil through CRISPR/Cas9-mediated mutagenesis of carotenoid cleavage dioxygenase 4. Transgenic Res. 27, 25–38. doi: 10.1007/s11248-017-0051-0, PMID: 29247330

[ref210] Welsch R. Arango J. Bär C. Salazar B. Al-Babili S. Beltrán J.. (2010). Provitamin a accumulation in cassava (Manihot esculenta) roots driven by a single nucleotide polymorphism in a phytoene synthase gene. Plant Cell 22, 3348–3356. doi: 10.1105/tpc.110.077560, PMID: 20889914 PMC2990137

[ref211] Welsch R. Zhou X. Yuan H. Álvarez D. Sun T. Schlossarek D.. (2018). Clp protease and OR directly control the proteostasis of phytoene synthase, the crucial enzyme for carotenoid biosynthesis in Arabidopsis. Mol. Plant 11, 149–162. doi: 10.1016/j.molp.2017.11.003, PMID: 29155321

[ref212] Wen L. Wang Y. Deng Q. Hong M. Shi S. He S.. (2020). Identifying a carotenoid cleavage dioxygenase (CCD4) gene controlling yellow/white fruit flesh color of “Piqiutao” (white fruit flesh) and its mutant (yellow fruit flesh). Plant Mol. Biol. Report. 38, 513–520. doi: 10.1007/s11105-020-01213-2

[ref213] Wilson F. M. Harrison K. Armitage A. D. Simkin A. J. Harrison R. J. (2019). CRISPR/Cas9-mediated mutagenesis of phytoene desaturase in diploid and octoploid strawberry. Plant Methods 15, 1–13. doi: 10.1186/s13007-019-0428-6 31068975 PMC6495592

[ref214] Wolabu T. W. Park J. J. Chen M. Cong L. Ge Y. Jiang Q.. (2020). Improving the genome editing efficiency of CRISPR/Cas9 in Arabidopsis and Medicago truncatula. Planta 252, 1–14. doi: 10.1007/s00425-020-03415-0 32642859 PMC7343739

[ref215] Wu S. Zhu H. Liu J. Yang Q. Shao X. Bi F.. (2020). Establishment of a PEG-mediated protoplast transformation system based on DNA and CRISPR/Cas9 ribonucleoprotein complexes for banana. BMC Plant Biol. 20, 1–10. doi: 10.1186/s12870-020-02609-8 32933485 PMC7493974

[ref216] Xing S. Miao J. Li S. Qin G. Tang S. Li H.. (2010). Disruption of the 1-deoxy-D-xylulose-5-phosphate reductoisomerase (DXR) gene results in albino, dwarf and defects in trichome initiation and stomata closure in Arabidopsis. Cell Res. 20, 688–700. doi: 10.1038/cr.2010.54, PMID: 20404857

[ref217] Xu Z. S. Feng K. Xiong A. S. (2019). CRISPR/Cas9-mediated multiply targeted mutagenesis in Orange and purple carrot plants. Mol. Biotechnol. 61, 191–199. doi: 10.1007/s12033-018-00150-6, PMID: 30644027

[ref218] Yan J. Kandianis C. B. Harjes C. E. Bai L. Kim E. H. Yang X.. (2010). Rare genetic variation at Zea mays crtRB1 increases Β-carotene in maize grain. Nat. Genet. 42, 322–327. doi: 10.1038/ng.551, PMID: 20305664

[ref219] Yan R. Wang Z. Ren Y. Li H. Liu N. Sun H. (2019). Establishment of efficient genetic transformation systems and application of crispr/cas9 genome editing technology in lilium pumilum dc. Fisch. and lilium longiflorum white heaven. Int. J. Mol. Sci. 20:2920. doi: 10.3390/ijms20122920 PMC6627044 31207994

[ref220] Yang X. Chen L. He J. Yu W. (2017). Knocking out of carotenoid catabolic genes in rice fails to boost carotenoid accumulation, but reveals a mutation in strigolactone biosynthesis. Plant Cell Rep. 36, 1533–1545. doi: 10.1007/s00299-017-2172-6, PMID: 28676963

[ref221] Yin K. Han T. Liu G. Chen T. Wang Y. Yu A. Y. L.. (2015). A geminivirus-based guide RNA delivery system for CRISPR/Cas9 mediated plant genome editing. Sci. Rep. 5, 1–10. doi: 10.1038/srep14926 PMC4598821 26450012

[ref222] Yoo H. J. Park W. J. Lee G. M. Oh C. S. Yeam I. Won D. C.. (2017). Inferring the genetic determinants of fruit colors in tomato by carotenoid profiling. Molecules 22, 1–14. doi: 10.3390/molecules22050764 PMC6154295 28481314

[ref223] Yuan D. Chen J. Shen H. Yang W. (2008). Genetics of flesh color and nucleotide sequence analysis of phytoene synthase gene 1 in a yellow-fruited tomato accession PI114490. Sci. Hortic. 118, 20–24. doi: 10.1016/j.scienta.2008.05.011

[ref224] Zhang F. LeBlanc C. Irish V. F. Jacob Y. (2017). Rapid and efficient CRISPR/Cas9 gene editing in citrus using the YAO promoter. Plant Cell Rep. 36, 1883–1887. doi: 10.1007/s00299-017-2202-4, PMID: 28864834

[ref225] Zhang B. Liu C. Wang Y. Yao X. Wang F. Wu J.. (2015). Disruption of a CAROTENOID CLEAVAGE DIOXYGENASE 4 gene converts flower colour from white to yellow in Brassica species. New Phytol. 206, 1513–1526. doi: 10.1111/nph.13335, PMID: 25690717

[ref226] Zhang B. Yang X. Yang C. Li M. Guo Y. (2016). Exploiting the CRISPR/Cas9 system for targeted genome mutagenesis in petunia. Sci. Rep. 6, 1–8. doi: 10.1038/srep20315 26837606 PMC4738242

[ref227] Zhang L. Zhan X. Wang X. Xu J. Wang M. Li L.. (2019a). SEED CAROTENOID DEFICIENT functions in isoprenoid biosynthesis via the plastid MEP pathway. Plant Physiol. 179, 1723–1738. doi: 10.1104/pp.18.01148, PMID: 30718347 PMC6446789

[ref228] Zhang S. Zhang R. Gao J. Gu T. Song G. Li W.. (2019b). Highly efficient and heritable targeted mutagenesis in wheat via the agrobacterium tumefaciens-mediated CRISPR/Cas9 system. Int. J. Mol. 20:4257. doi: 10.3390/ijms20174257 PMC6747105 31480315

[ref229] Zhang S. Zhang R. Gao J. Song G. Li J. Li W.. (2021). CRISPR/Cas9-mediated genome editing for wheat grain quality improvement. Plant Biotechnol. J. 19, 1684–1686. doi: 10.1111/pbi.13647 34143557 PMC8428824

[ref230] Zhang H. Zhang J. Wei P. Zhang B. Gou F. Feng Z.. (2014). The CRISPR/Cas9 system produces specific and homozygous targeted gene editing in rice in one generation. Plant Biotechnol. J. 12, 797–807. doi: 10.1111/pbi.12200, PMID: 24854982

[ref231] Zhao J. Fang Y. Kang S. Ruan B. Xu J. Dong G.. (2014). Identification and characterization of a new allele for ZEBRA LEAF 2, a gene encoding carotenoid isomerase in rice. S. Afr. J. Bot. 95, 102–111. doi: 10.1016/j.sajb.2014.08.011

[ref232] Zhao C. Safdar L. B. Xie M. Shi M. Dong Z. Yang L.. (2021). Mutation of the PHYTOENE DESATURASE 3 gene causes yellowish-white petals in Brassica napus. Crop J. 9, 1124–1134. doi: 10.1016/j.cj.2020.10.012

[ref233] Zhou X. Welsch R. Yang Y. Álvarez D. Riediger M. Yuan H.. (2015). Arabidopsis OR proteins are the major posttranscriptional regulators of phytoene synthase in controlling carotenoid biosynthesis. PNAS 112, 3558–3563. doi: 10.1073/pnas.1420831112 25675505 PMC4371912

[ref234] Zhou H. Yang M. Zhao L. Zhu Z. Liu F. Sun H.. (2021). HIGH-TILLERING and DWARF 12 modulates photosynthesis and plant architecture by affecting carotenoid biosynthesis in rice. J. Exp. Bot. 72, 1212–1224. doi: 10.1093/jxb/eraa497, PMID: 33097962

[ref235] Zhu J. Song N. Sun S. Yang W. Zhao H. Song W.. (2016). Efficiency and inheritance of targeted mutagenesis in maize using CRISPR-Cas9. J. Genet. Genomics 43, 25–36. doi: 10.1016/j.jgg.2015.10.006, PMID: 26842991

[ref236] Zhu C. Zheng X. Huang Y. Ye J. Chen P. Zhang C.. (2019). Genome sequencing and CRISPR/Cas9 gene editing of an early flowering mini-citrus (Fortunella hindsii). Plant Biotechnol. J. 17, 2199–2210. doi: 10.1111/pbi.13132, PMID: 31004551 PMC6790359

[ref237] Zou J. Zhang S. Zhang W. Li G. Chen Z. Zhai W.. (2006). The rice HIGH-TILLERING DWARF1 encoding an ortholog of Arabidopsis MAX3 is required for negative regulation of the outgrowth of axillary buds. Plant J. 48, 687–698. doi: 10.1111/j.1365-313X.2006.02916.x, PMID: 17092317

